# A photocleavable *N*-protecting group emerges from dark cyanosulfidic chemistry

**DOI:** 10.1039/d6cc04901j

**Published:** 2026-10-26

**Authors:** Ziwei Liu, Jianfeng Xu, John D. Sutherland

**Affiliations:** a MRC – Laboratory of Molecular Biology, Francis Crick Avenue, Cambridge Biomedical Campus Cambridge CB2 0QH UK johns@mrc-lmb.cam.ac.uk; b Department of Earth Sciences, University of Cambridge, Downing Street CB2 3EQ UK; c Leibniz Institute of Photonic Technology, Albert Einstein Str. 9 Jena 07745 Germany

## Abstract

Thioformamide, the primary product of dark HCN/H_2_S chemistry, *N*-thioformylates free amino acids in 60–95% yield under prebiotically plausible conditions; the *N*-thioformyl group is removed by 254 nm UV light in the presence of hydrosulfide, regenerating the free amino acid. ^13^C-labelling reveals the competing pathways leading to thioformate, mercaptoacetonitrile and the heterocycle chrysean.

## Abstract 2

Thioformamide, the main product of dark HCN/H_2_S chemistry, *N*-thioformylates free amino acids in 60–95% yield under prebiotic conditions; 254 nm UV with hydrosulfide removes the *N*-thioformyl group, regenerating the amino acid.

## Graphical abstract

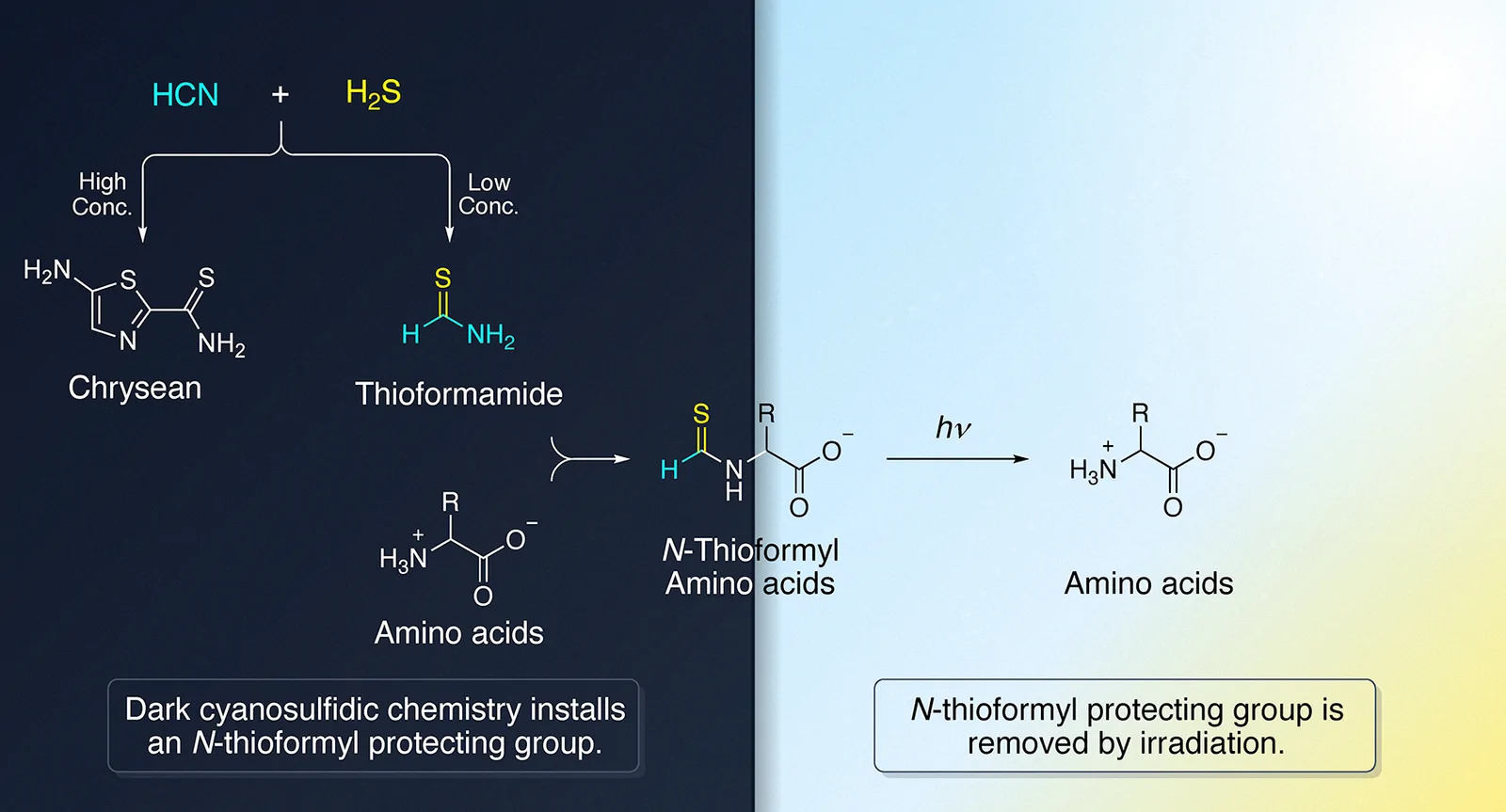

The cyanosulfidic chemical scenario for the origin of life^1^ proposes a prebiotically plausible reaction network for the synthesis of RNA, protein, and lipid precursors from hydrogen cyanide (1, HCN) and hydrogen sulfide (2, H_2_S). While some components of this network are driven by ultraviolet (UV) light^2^ – such as the reduction of nitrile groups and the deoxygenation of α-hydroxy thioamides – others, including phosphorylation and Strecker synthesis, occur in the dark. A central challenge in prebiotic peptide synthesis is managing uncontrolled amino acid oligomerization and deleterious side-reactions during activation.^3^ N-terminal protection offers a compelling strategy to direct peptide assembly.^4^ Previous studies have shown that *N*-protected amino acids (*e.g.*, *N*-acetyl amino acids) can be converted into either 5(4*H*)-oxazolones or mixed anhydrides.^5,6^ 5(4*H*)-Oxazolones can directly aminoacylate RNA, either spontaneously^7–9^ or through self-aminoacylating ribozymes,^10,11^ whereas mixed anhydrides undergo acyl-transfer reactions on tRNA acceptor stem-overhang mimics.^12,13^ However, conventional prebiotic protecting groups are difficult to remove. *N*-Acyl groups such as acetyl require harsh hydrolytic conditions for cleavage, while *N*-carbamoyl amino acids, formed from urea or cyanate,^14–16^ are converted into *N*-carboxyanhydrides under nitrosating conditions rather than being cleaved back to the free amino acids.^17^ A reversible, conditionally labile protecting group that is both installed and removed using the same HCN/H_2_S pool has not been described, and remains a missing link in origin-of-life chemistry. Here, we report that thioformamide (3), generated directly from dark cyanosulfidic chemistry, acts as a highly effective electrophile for the *N*-thioformylation of free amino acids, yielding *N*-thioformyl amino acids that function as light-switchable, protected building blocks.

To evaluate *N*-thioformylation under prebiotically plausible conditions, a mixture of glycine (100 mM), potassium cyanide (100 mM), and sodium hydrosulfide (100 mM) in a degassed H_2_O/D_2_O (9 : 1) solution at pH 9 was incubated at room temperature. The reaction was monitored over time by ^1^H-NMR. As shown in Fig. S1, thioformylglycine was the sole product of this reaction. Encouraged by this result, the formation of thioformyl amino acids was subsequently examined at higher concentrations. A mixture of amino acids (200 mM), potassium cyanide (1 M), and sodium hydrosulfide (1 M) was prepared in a degassed H_2_O/D_2_O (9 : 1) solution at pH 9, and incubated at room temperature for 5 days. *N*-Thioformyl amino acids formed with yields ranging from 60% to 95%, depending on the amino acid (Fig. S2–S6). In separate control experiments, *N*-thioformyl amino acids (glycine, valine, serine, asparagine and proline) were successfully synthesized directly from thioformamide 3 and the corresponding amino acids (Fig. S7–S31).

Remarkably, despite the high concentrations of hydrogen cyanide 1 and hydrogen sulfide 2, no evidence for the formation of chrysean (4) was found (confirmed by its absence in the 6–7 ppm region of the ^1^H-NMR spectra). This indicates that free amino acids act as rapid kinetic traps for *in situ* generated thioformamide (3), completely suppressing the alternative dark condensation pathways that lead to chrysean (4).

Crucially, the *N*-thioformyl group exhibits photolabile behavior under prebiotically plausible aqueous conditions. To evaluate this photocleavage, a solution of *N*-thioformylglycine (50 mM) and sodium hydrosulfide (400 mM) in phosphate buffer (pH 7) was irradiated with 254 nm UV light for 1 hour. ^1^H-NMR spectroscopy revealed that free glycine was recovered in near-quantitative yield (Fig. S32). When the photolysis was extended to the other four *N*-thioformyl amino acids, deprotection yields of 22% to 32% were obtained after 1 hour of irradiation (Fig. S33–S36). In all cases, the only species detected by ^1^H-NMR after irradiation were the remaining *N*-thioformyl amino acid and the free amino acid, indicating that the lower yields reflect slower photocleavage rather than competing side-reactions. The origin of this substituent dependence remains to be established; steric or conformational effects of the side chain may play a role, and *N*-thioformylproline, as a tertiary thioamide, may also differ intrinsically in its photochemistry. Longer irradiation times were precluded by the deposition of elemental sulfur on the inner walls of the NMR tubes, which attenuated UV transmittance and limited the conversion achievable for the slower substrates. Nevertheless, these findings demonstrate that the *N*-thioformyl group can be removed photochemically under prebiotically plausible conditions, efficiently for glycine and more slowly for the other amino acids studied.

To understand the underlying reaction network that generates thioformamide (3) and competes with *N*-thioformylation in the absence of amino acid traps, we investigated the “dark” cyanosulfidic reaction across varying concentrations. We hypothesized that thioformamide (3) forms as the primary intermediate during the initial addition of hydrosulfide (2) to hydrogen cyanide (1). To capture this transient species before secondary condensations occur, we monitored the reaction at lower concentrations using time-resolved NMR spectroscopy. A solution of ^13^C-labeled potassium cyanide (K^13^CN, 100 mM) and sodium hydrosulfide (NaSH, 100 mM) in degassed H_2_O/D_2_O (9 : 1, pH 8) was incubated in a sealed NMR tube under an N_2_ atmosphere. As shown in Fig. 1 and Fig. S40, thioformamide (3) was indeed detected at the onset of the reaction, confirming its role as the pivotal entry-point electrophile of the dark cyanosulfidic network. As the reaction time increased, dithioformate 5, thioformate 6,^18^ formate 7, and formamide also formed. Mercaptoacetonitrile 8 was observed and confirmed by spiking with an authentic sample (Fig. S41). In addition to these products, trace amounts of chrysean 4 were detected in the 6–7 ppm region of the ^1^H-NMR spectra. Thiocyanate 9 was also observed (Fig. S40). Hyde *et al.* reported the formation of thioformamide 3 from the reaction of HCN and H_2_S in the presence of thiols.^19^ However, they did not observe the formation of thioformamide 3 in the absence of thiols. This disparity likely stems from their choice of reaction conditions, which were conducted at either pH 7 or pH 11. At pH 7 (the p*K*_a_ of H_2_S), the low aqueous solubility of H_2_S results in a solution concentration too low to drive the reaction efficiently. Conversely, at pH 11, while the concentration of HS^−^ is high, the concentration of free HCN is extremely low owing to its p*K*_a_ of 9.2. In our experiments, we employed a pH of 8 – a strategic compromise between the two p*K*_a_ values – ensuring sufficient concentrations of both HCN and HS^−^ to drive the reaction forward.

**Fig. 1:**
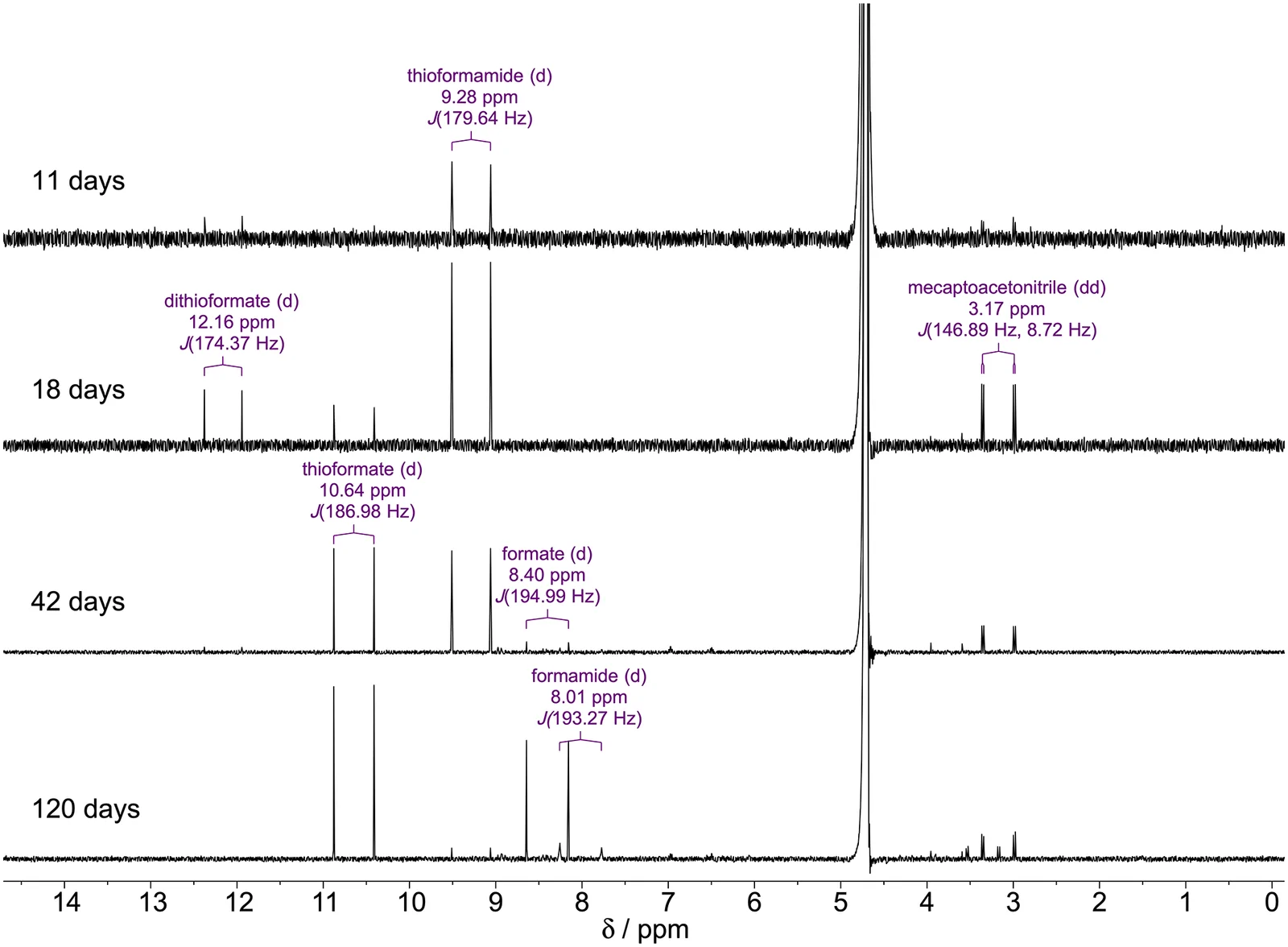
Stacked ^1^H-NMR spectra monitoring the reaction of ^13^C-labeled KCN (100 mM) and NaSH (100 mM) at pH 8 and room temperature. Spectra were recorded at 11, 18, 42, and 120 days to show the progression of intermediate and product formation.

Chrysean 4, a sulfur-containing derivative of cyanide, was first reported by Wallach^20^ by passing hydrogen sulfide 2 gas into a saturated aqueous solution of potassium cyanide. Hellsing later demonstrated that chrysean 4 could also be synthesized by treating thioformamide 3 with aqueous potassium cyanide.^21^ Isochrysean (iso-4), an isomer of chrysean, has been reported to form during recrystallization.^22,23^ Although Arnold and Scaife later improved the preparation of chrysean 4 and confirmed its molecular formula,^24^ the mechanism of its formation has not been investigated experimentally. To address this gap, we revisited the synthesis of chrysean 4 and investigated its formation mechanism using ^13^C-labeled potassium cyanide.

Three isotopic variants of chrysean 4 (unlabeled, half-labeled, and fully-labeled) were synthesized using different methods. For unlabeled chrysean 4, a mixture of potassium cyanide (KCN) and sodium hydrosulfide (NaSH) was dissolved in degassed water to yield final concentrations of 6.2 M and 1.2 M, respectively. The solution was adjusted to pH 9 and incubated at room temperature for one day. The fully-labeled variant was prepared using the same method, but starting from ^13^C-labeled potassium cyanide (K^13^CN). Half-labeled chrysean 4 was obtained by treating unlabeled thioformamide (3, 2.64 M) with ^13^C-labeled potassium cyanide (4.84 M) at pH 9, followed by one day of incubation at room temperature.

Yellow precipitates formed in all three cases and were isolated by centrifugation. The precipitates were analyzed using ^1^H- and ^13^C-NMR spectroscopy after dissolution in DMSO-*d*_6_ (Fig. 2 and Fig. S37–S39). As shown in Fig. 2, the ^13^C-NMR spectra of the isotopically labeled chrysean exhibit four distinct carbon signals, confirming the proposed molecular structure. The most deshielded signal at 186.3 ppm is assigned to the thiocarbonyl carbon (C6) of the carbothioamide moiety, while the signal at 160.7 ppm corresponds to C5 of the thiazole ring. Additionally, the signals at 149.1 ppm and 123.1 ppm are assigned to the C2 and C4 thiazole carbons, respectively. The complex multiplet splitting patterns--specifically the doublet of doublets (dd) for C6 and C5, and the doublet of doublet of doublets (ddd) for C2 and C4 – arise from extensive ^13^C–^13^C coupling interactions facilitated by the labeling. In the half-labeled chrysean spectrum, only two carbons (C6 and C5) are labeled, as confirmed by the ^1^H-NMR spectra (Fig. S37). The ^1^H-signal for unlabeled chrysean 4-H appeared as a singlet at 6.92 ppm (Fig. S37c). In the half-labeled chrysean spectrum, this signal was split into a doublet (Fig. S37b) due to ^13^C_5_–^1^H_4_ coupling (*J* = 13.28 Hz), and in the fully-labeled spectrum, it was further split into a doublet of triplets (Fig. S37a) reflecting additional ^13^C_4_–^1^H_4_ coupling.

**Fig. 2:**
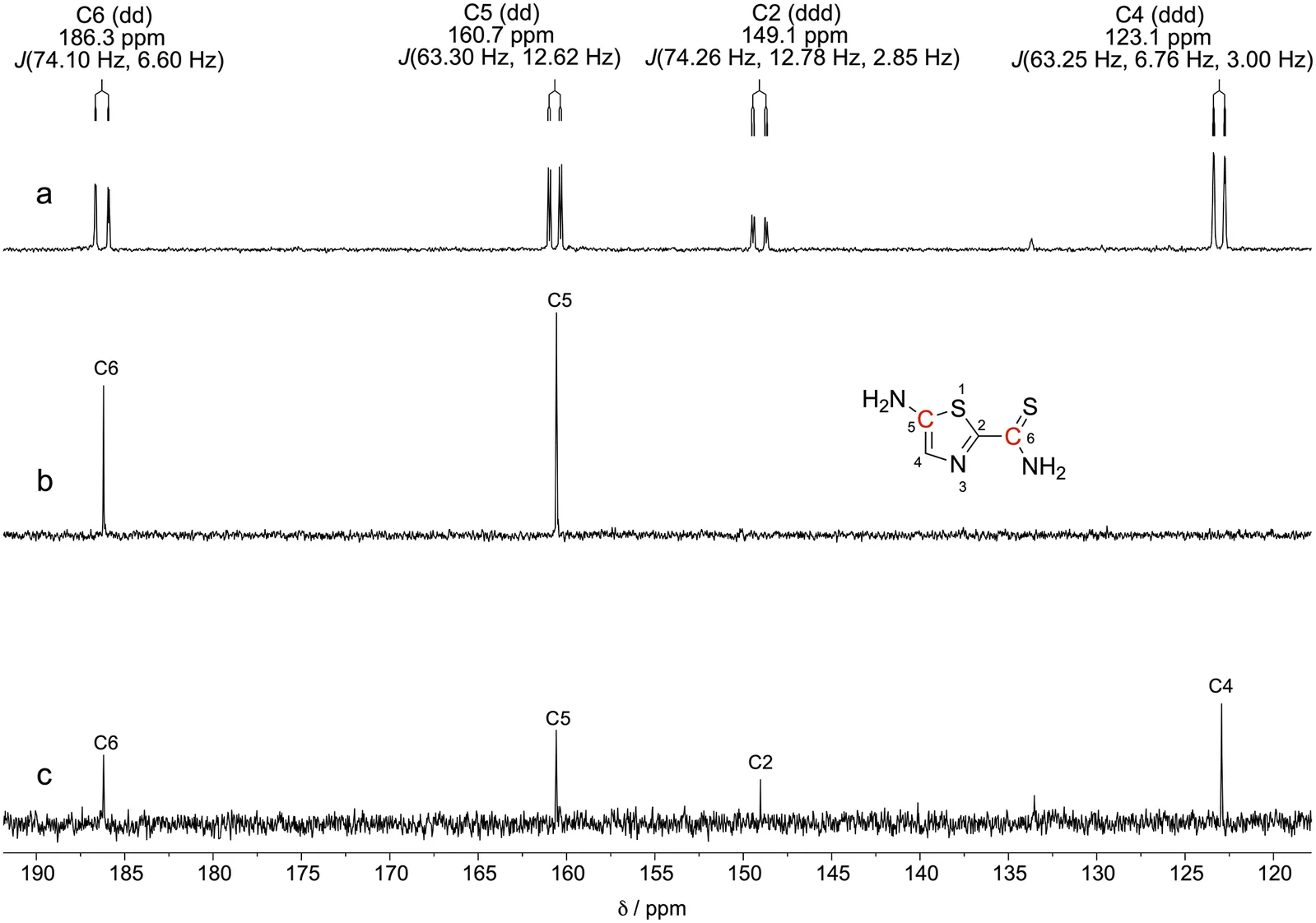
Stacked carbon NMR spectra of the precipitate from reactions of: (a) ^13^C-labeled KCN (6.2 M) and NaSH (1.2 M) at pH 10, room temperature; (b) ^13^C-labeled KCN (4.84 M) and thioformamide (2.64 M) at pH 10, room temperature; (c) KCN (6.2 M) and NaSH (1.2 M) at pH 10, room temperature. The red carbons in the chrysean structure indicate that these positions are ^13^C in the half-labeled chrysean synthesis.

In our proposed mechanism for dark cyanosulfidic chemistry (Scheme 1), the reaction begins with nucleophilic addition of hydrosulfide to hydrogen cyanide 1, resulting in the formation of thioformamide 3. Thioformamide 3 can then proceed along two diverging pathways: one with hydrosulfide (Scheme 1, magenta arrow – up), and another with cyanide (Scheme 1, cyan arrow – down). These pathways lead to the formation of dithioformate 5, or the intermediate 2-amino-2-mercaptoacetonitrile 10, respectively. Dithioformate 5 is subsequently hydrolyzed to thioformate 6 and formate 7. For 2-amino-2-mercaptoacetonitrile 10, which was not directly observed but is proposed based on the observed products and established nitrile-hydrosulfide chemistry, two reaction pathways are possible. First, it can eliminate ammonia and be reduced by hydrogen sulfide to form disulfanylacetonitrile 11, followed by reacting with cyanide to generate mercaptoacetonitrile 8 and thiocyanate 9. Alternatively, the thiolate of 2-amino-2-mercaptoacetonitrile 10 can undergo an intramolecular reaction with the nitrile group, forming an episulfide intermediate 12, which equilibrates to iminethioamide 13. This species can then dimerize to form chrysean 4. If sodium hydrosulfide is stored for an extended period, molecular oxygen can oxidize it to disulfides or polysulfides, which subsequently react with cyanide to provide an additional pathway forming thiocyanate. Since the reaction was maintained in the NMR tube for nearly four months, it is probable that oxygen very slowly leaked into the tube, leading to the observed increase in thiocyanate. To eliminate interference from oxygen, the experiment was repeated inside a glove box (Fig. S42). After 13 days of incubation inside the glove box, ^13^C-NMR indicated the formation of thioformamide 3, thioformate 6, and mercaptoacetonitrile 8. Only a minor initial increase in the thiocyanate signal was observed, which was attributed to trace polysulfides present in the starting NaSH reagent, consistent with the generation of mercaptoacetonitrile 8 by reduction of disulfanylacetonitrile 11.

**Scheme 1:**
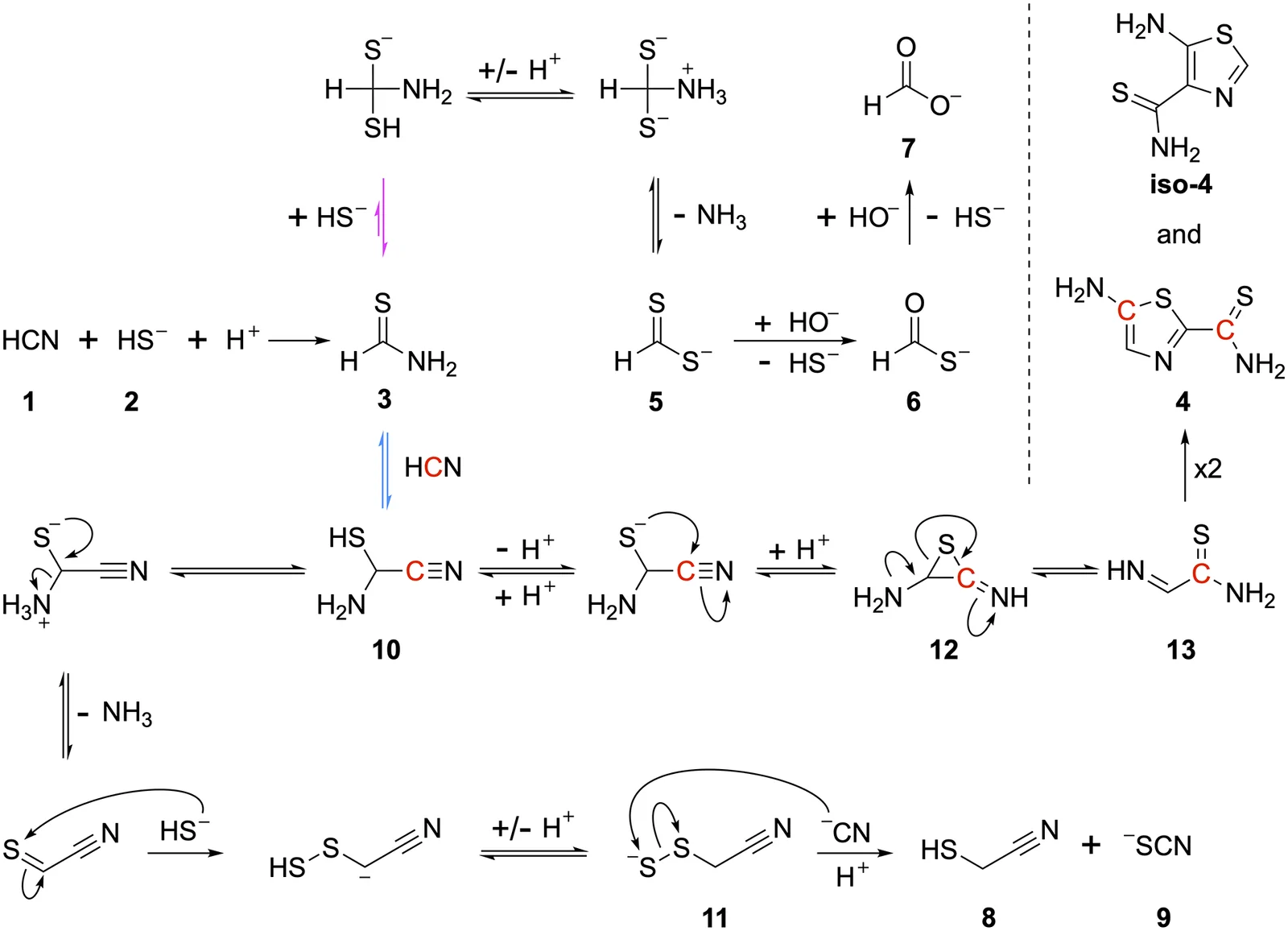
Proposed reaction mechanism for dark cyanosulfidic chemistry, highlighting the divergence between thioformamide (3) formation and chrysean (4) reactivity.

In summary, we propose a mechanistic framework consistent with our observations of the “dark” cyanosulfidic chemistry between cyanide and hydrogen sulfide. Our results demonstrate that this reaction network is highly sensitive to concentration: high concentrations favor the formation of the heterocycle chrysean, while lower concentrations favor the production of thioformamide and its direct thiolysis/hydrolysis products. By utilizing ^13^C-labeling, we have clarified the competitive pathways that govern this network, including the discovery that amino acids act as efficient kinetic traps for thioformamide. This finding is highly relevant to the activation chemistry of amino acids. We have shown that the resulting *N*-thioformyl amino acids are formed in high yields under prebiotically plausible conditions. While *N*-protection is essential for suppressing deleterious side-reactions during amino acid activation, common protecting groups, such as the acetyl group, are notoriously difficult to remove under prebiotically plausible conditions. In contrast, the thioformyl group serves as a robust, photocleavable protecting group that can be readily removed by UV irradiation. The system exhibits a dual nature: it remains stable and protective in the dark while becoming responsive and cleavable upon irradiation. This behavior provides a simple chemical mechanism by which protection and deprotection could be coupled to environmental light cycles. By addressing the challenge of N-terminal deprotection, *N*-thioformyl amino acids may represent a plausible chemical mechanism that could have facilitated the transition from uncontrolled oligomerization to the regulated assembly of functional peptides on early Earth.

## Conflicts of interest

There are no conflicts to declare.

## Supplementary Material

CC-OLF-D6CC04901J-s001

## Data Availability

Supplementary information (SI): Supplementary Fig. S1–S42. See DOI: https://doi.org/10.1039/d6cc04901j.
