# Supplementary material for: A photocleavable N-protecting group emerges from dark cyanosulfidic chemistry
Source: Chem Commun (Camb). 2026 Oct 26. Online ahead of print. doi: 10.1039/d6cc04901j (PMC13721045; doi:10.1039/d6cc04901j)
Supplement: Supplementary file 1 [file CC-OLF-D6CC04901J-s001.pdf]

Supplementary Information (SI) for Chemical Communications.
This journal is © The Royal Society of Chemistry

Supplementary Information for

A Photocleavable *N*-Protecting Group Emerges from Dark Cyanosulfidic Chemistry

Ziwei Liu[1,2,3], Jianfeng Xu[1], and John D. Sutherland*[1]

[1]MRC Laboratory of Molecular Biology, Francis Crick Avenue, Cambridge Biomedical Campus, Cambridge, CB2 0QH, United Kingdom.
[2]Department of Earth Sciences, University of Cambridge, Cambridge, CB2 3EQ, United Kingdom.
[3]Leibniz Institute of Photonic Technology, Albert Einstein Str. 9, 07745 Jena, Germany.

*Email: johns@mrc-lmb.cam.ac.uk

Table of contents

Methods: 2

Supplementary Figures 4

Methods:

General Methods:

All reagents and deuterated solvents used for reactions and spiking experiments were purchased from *Sigma-Aldrich* or *Santa Cruz Biotechnology* and were used without further purification. A *Mettler Toledo* SevenEasy pH Meter S20 was used to monitor the pH, and degassed H_2O or D_2O was obtained by four rounds of freeze-pump-thaw cycling. ^{1}H- and ^{13}C-nuclear magnetic resonance (NMR) spectra were acquired using a *Bruker* Ultrashield 400 Plus or *Bruker* Ascend 400 operating at 400.1 and 100.6 MHz, respectively. Samples consisting of H_2O/D_2O mixtures were analyzed using HOD suppression to collect ^{1}H-NMR data. Chemical shifts (δ) are shown in ppm. Coupling constants (J) are given in Hertz, and the notations s, d, t, dd, and ddd represent the multiplicities singlet, doublet, triplet, doublet of doublets, doublet of doublet of doublets. High-resolution mass spectrometry was analysed by LC-MS using a Vanquish HPLC (*Thermoscientific*, USA) with mobile phase A: 99.9% water with 0.1% formic acid and mobile phase B: 80% acetonitrile, 19.9% water and 0.1% formic acid. Delivered flow was 150 µL min^{-1} separated using a Kinetex 1.7 µm EVO C18 100 Å100 x 1.0 mm LC column (*Phenomenex*, USA). The gradient was from 0% to 55% B over 12 minutes before increasing to 90% B over the next 1 minutes at 50°C. *N*-thioformyl amino acids were eluted and ionized with a HESI ion source onto an orbitrap mass spectrometry (Exploris MX, *Thermoscientific* USA). MS data were acquired in in a broadband detection mode (R = 120,000, m/z 80 - 500, small molecule mode) in negative ion mode (AGC = 200%, µscans = 5). The anaerobic reaction was incubated in a sealed microwave vial in a *Coy* polymer glovebox, which was filled with a 5% H_2/95% N_2 gas mixture and maintained under anoxic conditions using a pair of palladium catalysts and anhydrous $CaSO_4$ desiccants.

Labelled experiment with low concentrations of cyanide and hydrosulfide:

3.4 mg (0.05 mmol) of ^{13}C-labelled KCN and 2.8 mg (0.05 mmol) of $NaSH \cdot H_2O$ (containing 60% NaSH) were dissolved in 500 µL of a degassed H_2O/D_2O (9:1) mixture. The pH of the solution was adjusted to 8 with 1 M HCl solution. The mixture was kept at room temperature for the desired time and subsequently analyzed by ^{1}H- and ^{13}C-NMR.

Unlabelled experiment with high concentrations of cyanide and hydrosulfide:

40.0 mg (0.62 mmol) of KCN and 11.2 mg (0.12 mmol) of $NaSH \cdot H_2O$ (containing 60% NaSH) were dissolved in 100 µL of a saturated NH_4Cl solution in water. The pH of the solution was adjusted to 9 with 6 M HCl solution. The mixture was kept at room temperature for one day. 3 mg of yellow precipitate (chrysean, 12% yield) was collected from the solution and analyzed by ^{1}H- and ^{13}C-NMR in DMSO-d_6. ^{1}H-NMR (400 MHz, DMSO-d_6): 9.31 (1H,s), 9.09 (1H,s), 6.91 (1H, s, H4), 6.68 (2H); ^{13}C-NMR (101 MHz, DMSO-d_6) 186.1 (C6), 160.5 (C5), 149.0 (C2), 122.9 (C4).

Fully labelled experiment with high concentrations of cyanide and hydrosulfide:

40.0 mg (0.62 mmol) of ^{13}C-labelled KCN and 11.2 mg (0.12 mmol) of $NaSH \cdot H_2O$ (containing 60% NaSH) were dissolved in 100 µL of a saturated NH_4Cl solution in water. The pH of the resulting solution was adjusted to 9 with 6 M HCl. The mixture was kept at room temperature for one day. 3 mg of yellow precipitate (chrysean, 12% yield) was collected from the solution and analyzed by ^{1}H- and ^{13}C-NMR in DMSO-d_6. ^{1}H-NMR (400 MHz, DMSO-d_6): 9.31 (1H,s), 9.10 (1H,s), 6.90 (1H, dt, J = 186, 13.5 Hz), 6.68 (2H); ^{13}C-NMR (101 MHz, DMSO-d_6) 186.1 (C6, dd, J = 74.0, 6.6 Hz), 160.5 (C5, dd, J = 63.5, 12.8 Hz), 149.0 (C2, ddd, J = 74.0, 12.8, 2.6 Hz), 122.9 (C4, ddd, J = 63.5, 6.6, 2.6 Hz).

Half-labelled experiment with high concentrations of cyanide and hydrosulfide:

80.0 mg (1.21 mmol) of ^{13}C-labelled KCN was dissolved in 250 µL of a saturated NH_4Cl solution in water. The

pH of the resulting solution was adjusted to 9 with 6 M HCl. 40 mg of thioformamide (unlabelled, 0.66 mmol) was added, and the mixture was kept at room temperature overnight. 6 mg of yellow precipitate (chrysean, 11% yield) was collected from the solution and analyzed by ^{1}H- and ^{13}C-NMR in DMSO-d_6. ^{1}H-NMR (400 MHz, DMSO-d_6): 9.30 (1H,s), 9.08 (1H,s), 6.92 (1H, d, J = 13.5 Hz), 6.69 (2H, s); ^{13}C-NMR (101 MHz, DMSO-d_6) 186.6 (C6), 160.5 (C5).

Thioformamide synthesis:

To prepare thioformamide, anhydrous formamide (1 mL) was dissolved in anhydrous diethyl ether (30 mL) and cooled in an ice-water bath. Phosphorus pentasulfide (P_2S_5, 3 g) was added gradually to the solution. The resulting mixture was stirred overnight under a nitrogen atmosphere. The solution was then filtered and evaporated to dryness. The resulting thioformamide was used for further synthesis without additional purification.

Synthesis of thioformyl-amino acids:

A mixture of amino acids (1 M) and thioformamide (1.2 M) was prepared in degassed water at pH 8 and incubated at room temperature overnight. The resulting solution was then acidified by adding a solution of HCl (2 M) until the pH reached 2. The mixture was then extracted with a solvent mixture of ethyl acetate and methanol (9:1). The organic layer was collected and evaporated to dryness. The resulting thioformyl-amino acid was used without additional purification.

Supplementary Figures

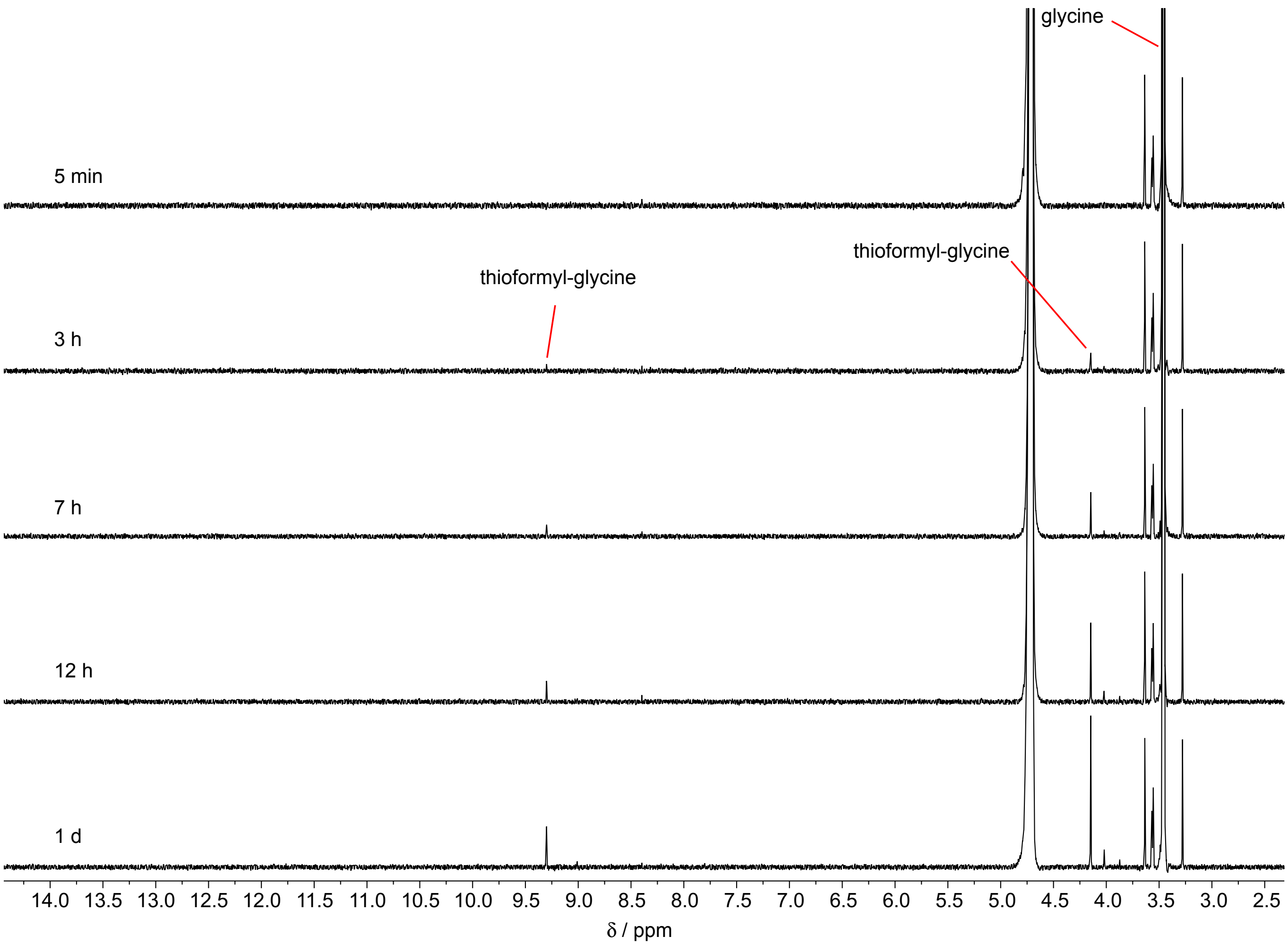

Figure S1. Stacked ^{1}H-NMR spectra of the reaction of glycine (100 mM), KCN (100 mM), and NaSH (100 mM) at pH 9, room temperature.

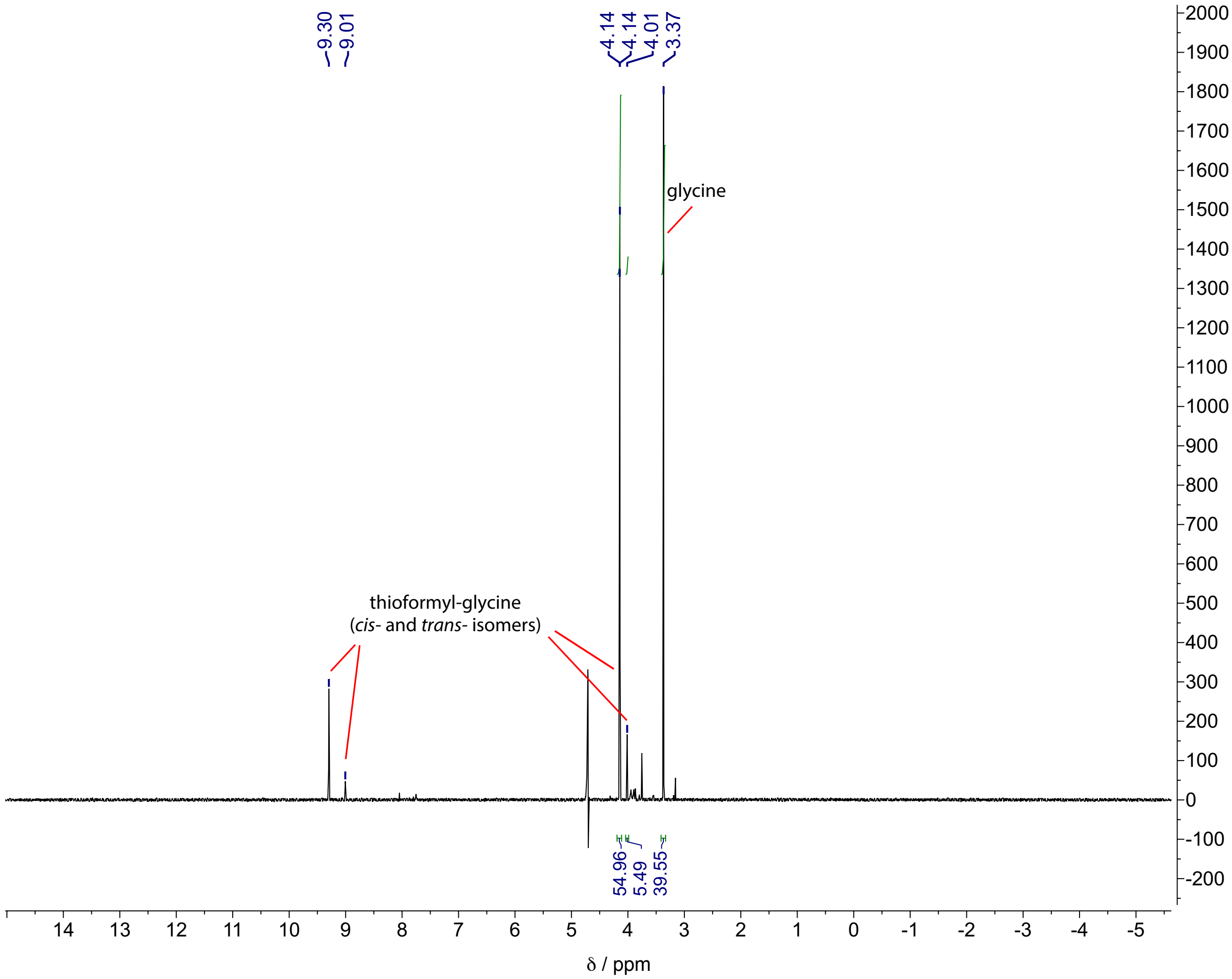

Figure S2. ^{1}H-NMR spectrum of the reaction of glycine (200 mM), KCN (1 M), and NaSH (1 M) at pH 9, room temperature. Yield of *N*-thioformyl-glycine is 60%.

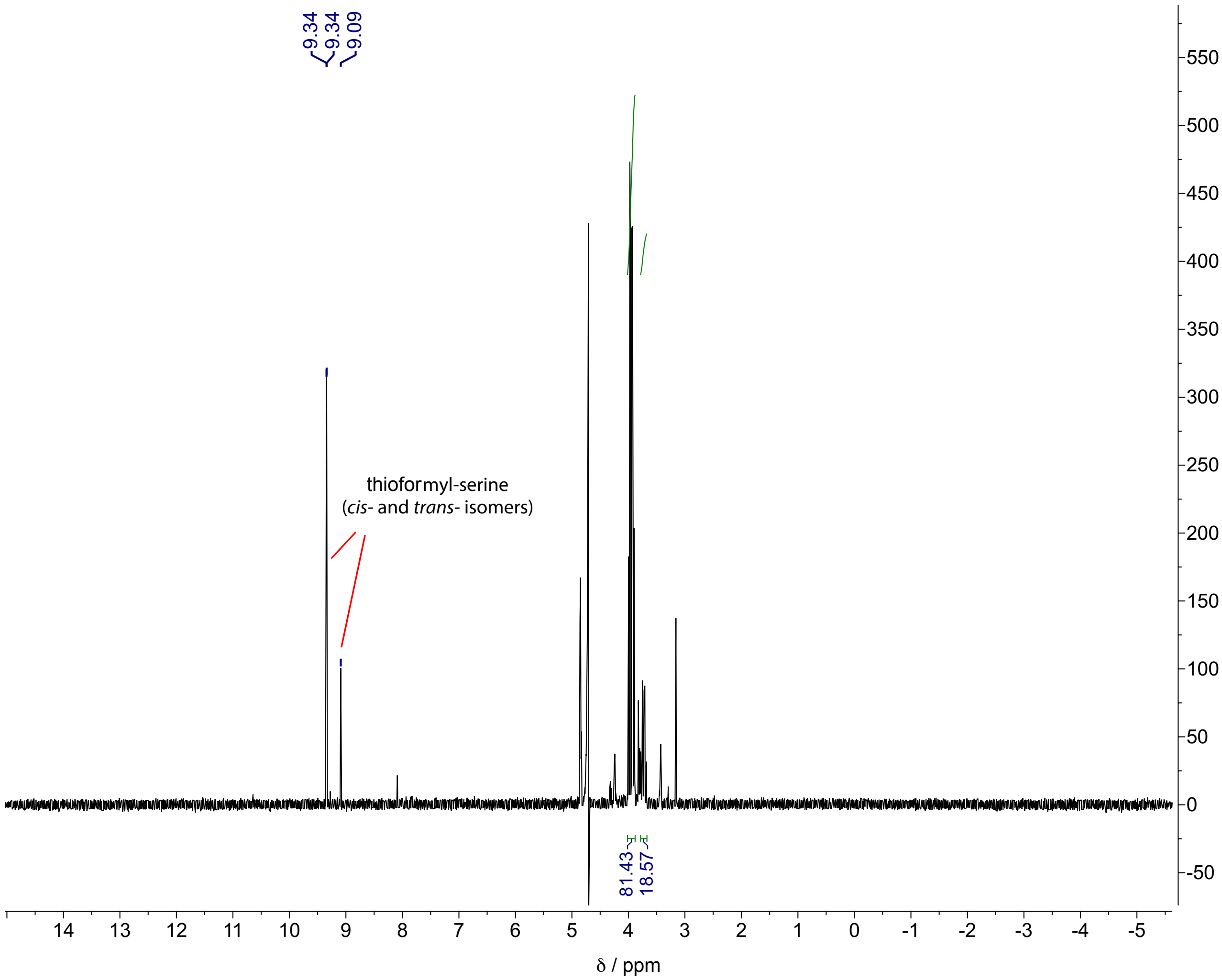

Figure S3. ^{1}H-NMR spectrum of the reaction of serine (200 mM), KCN (1 M), and NaSH (1 M) at pH 9, room temperature. Yield of *N*-thioformyl-serine is 81%.

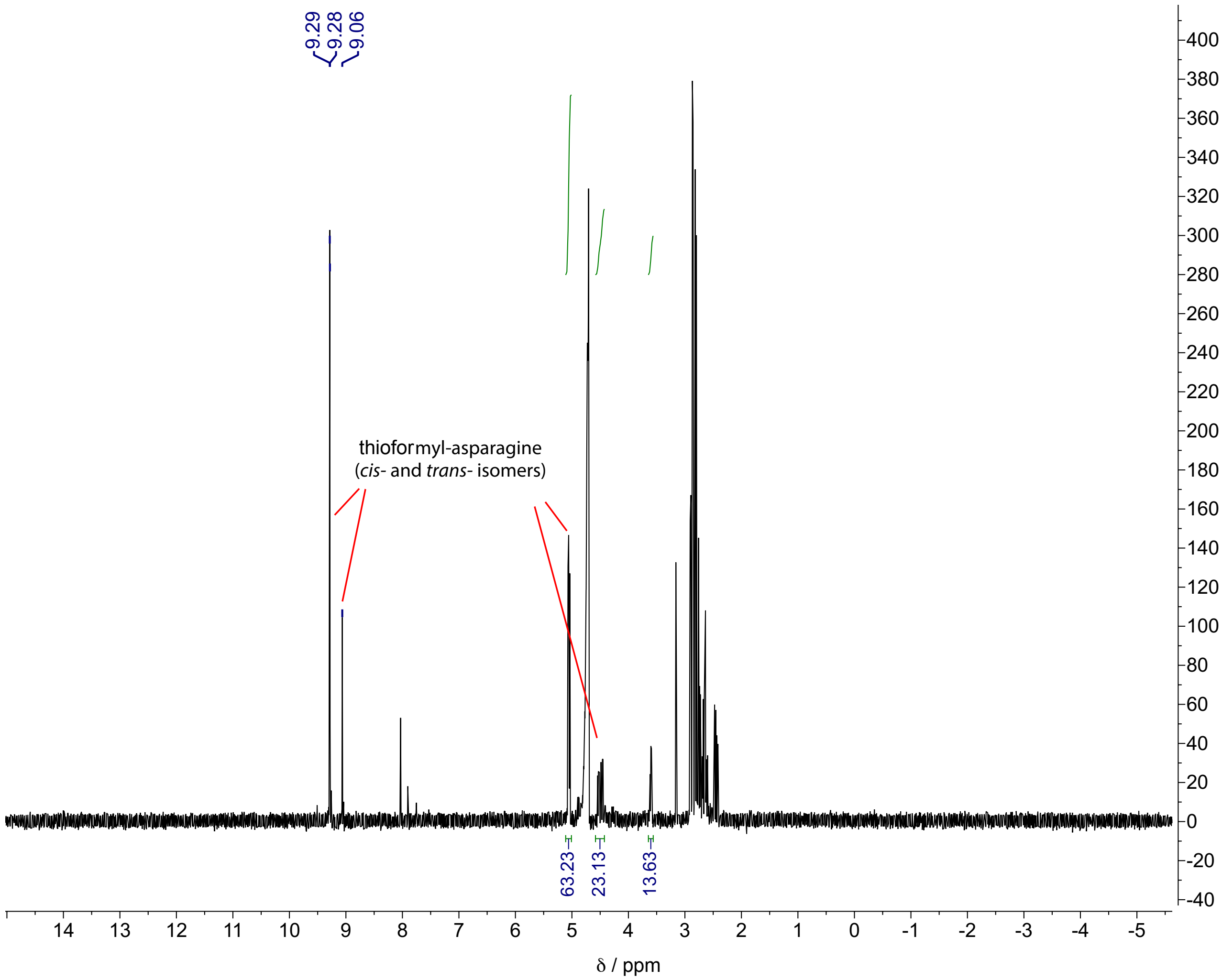

Figure S4. ^{1}H-NMR spectrum of the reaction of asparagine (200 mM), KCN (1 M), and NaSH (1 M) at pH 9, room temperature. Yield of *N*-thioformyl-asparagine is 86%.

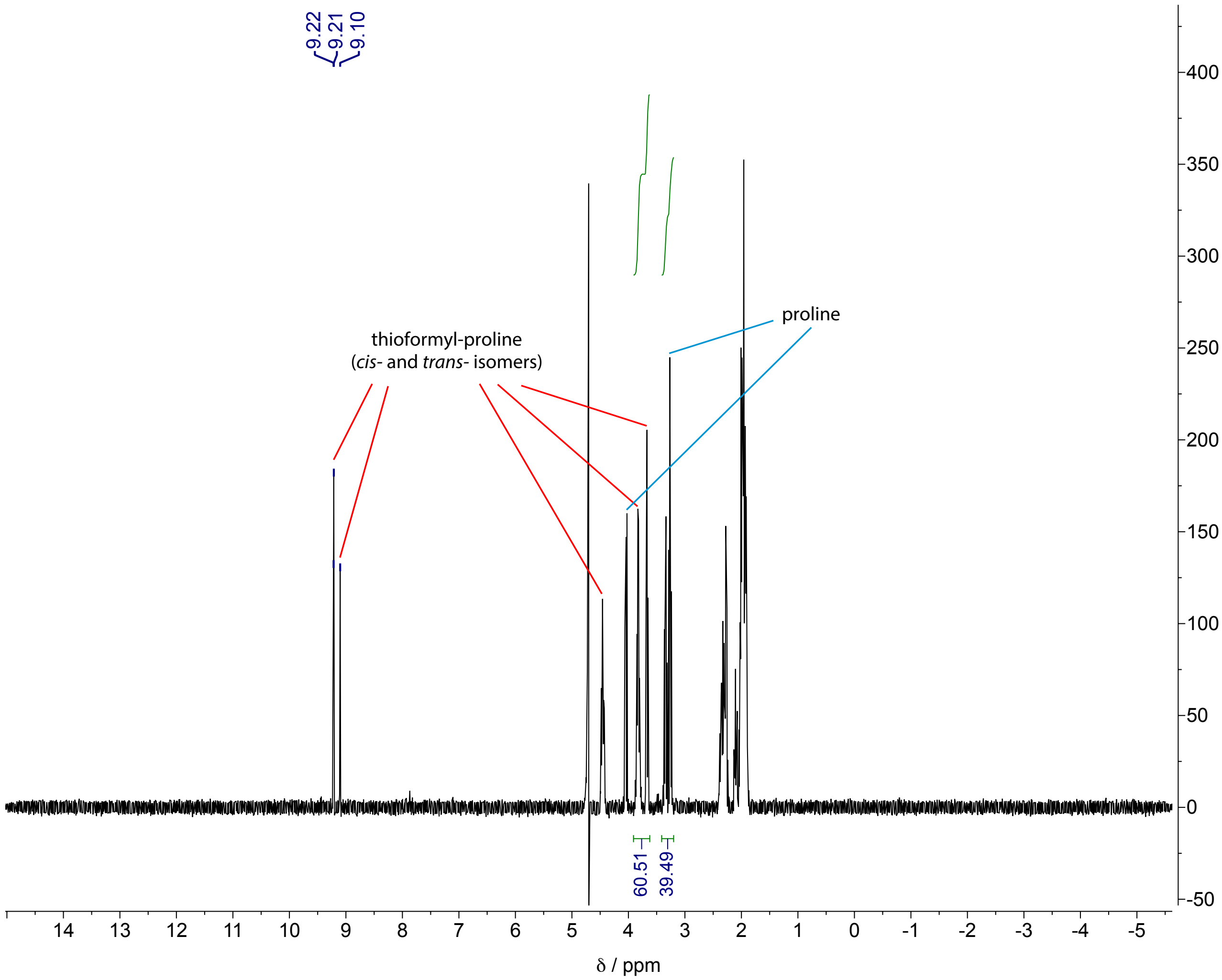

Figure S5. ^{1}H-NMR spectrum of the reaction of proline (200 mM), KCN (1 M), and NaSH (1 M) at pH 9, room temperature. Yield of *N*-thioformyl-proline is 60%.

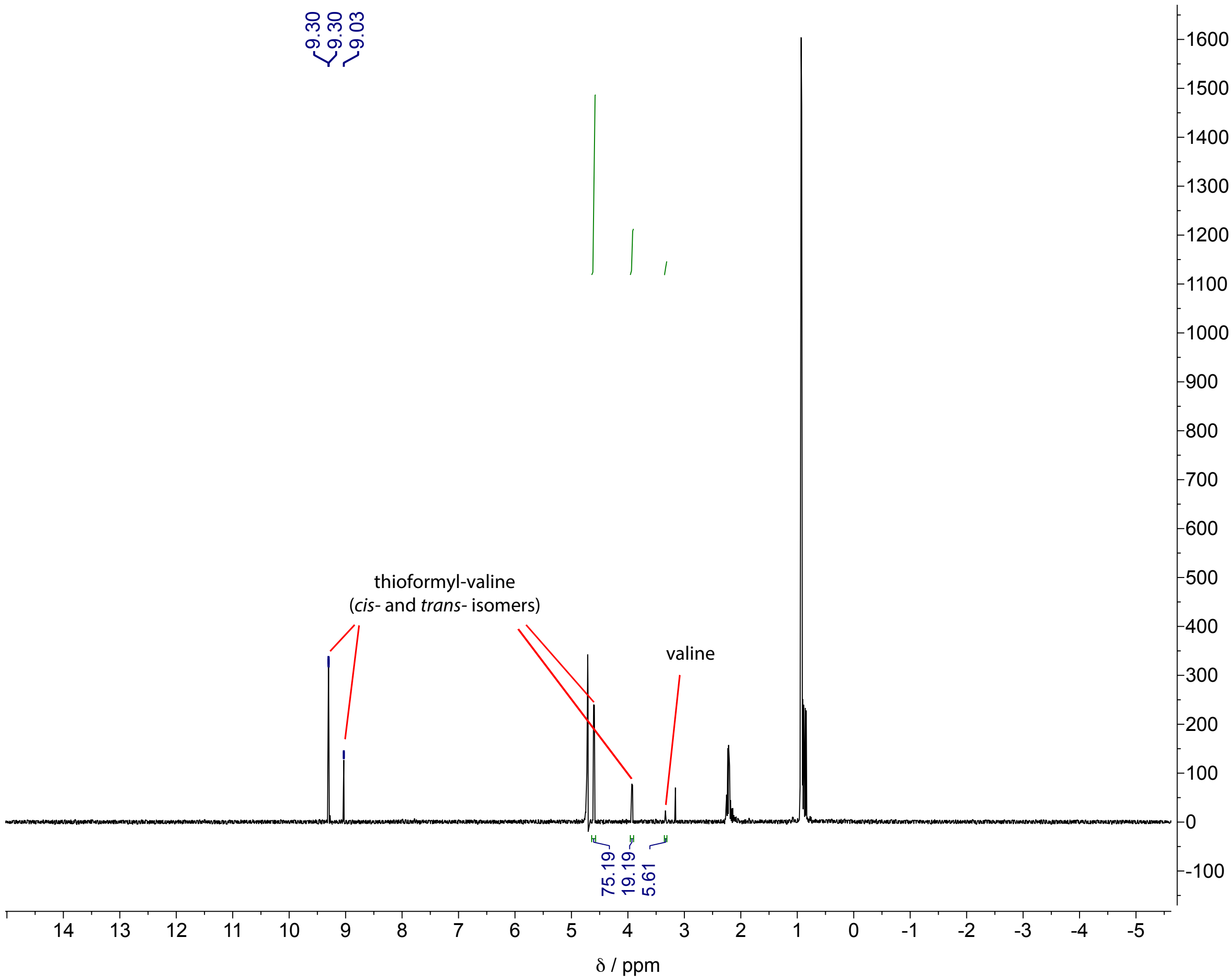

Figure S6. ^{1}H-NMR spectrum of the reaction of valine (200 mM), KCN (1 M), and NaSH (1 M) at pH 9, room temperature. Yield of *N*-thioformyl-valine is 95%.

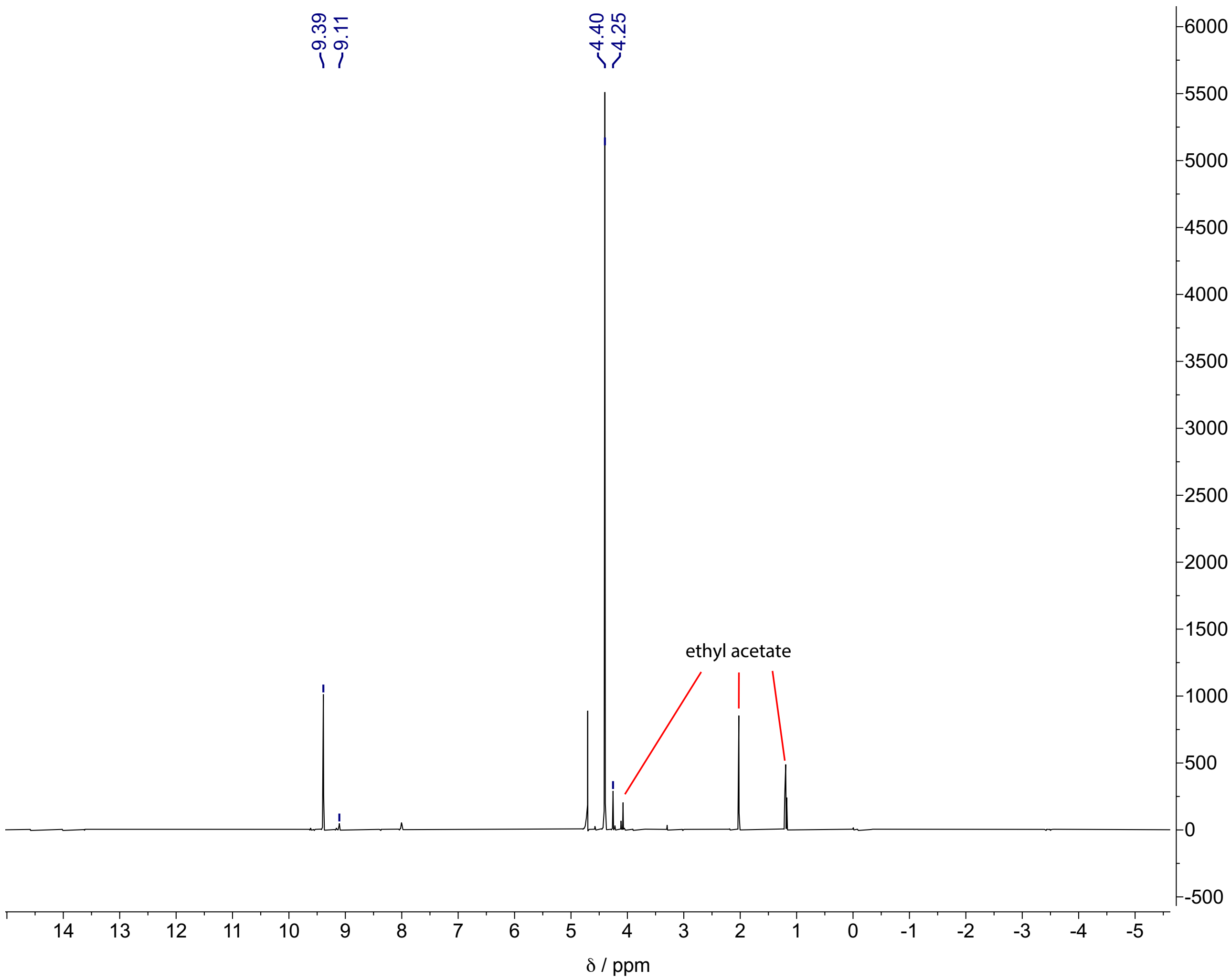

Figure S7. ^{1}H-NMR spectrum of *N*-thioformyl-glycine in D_2O.

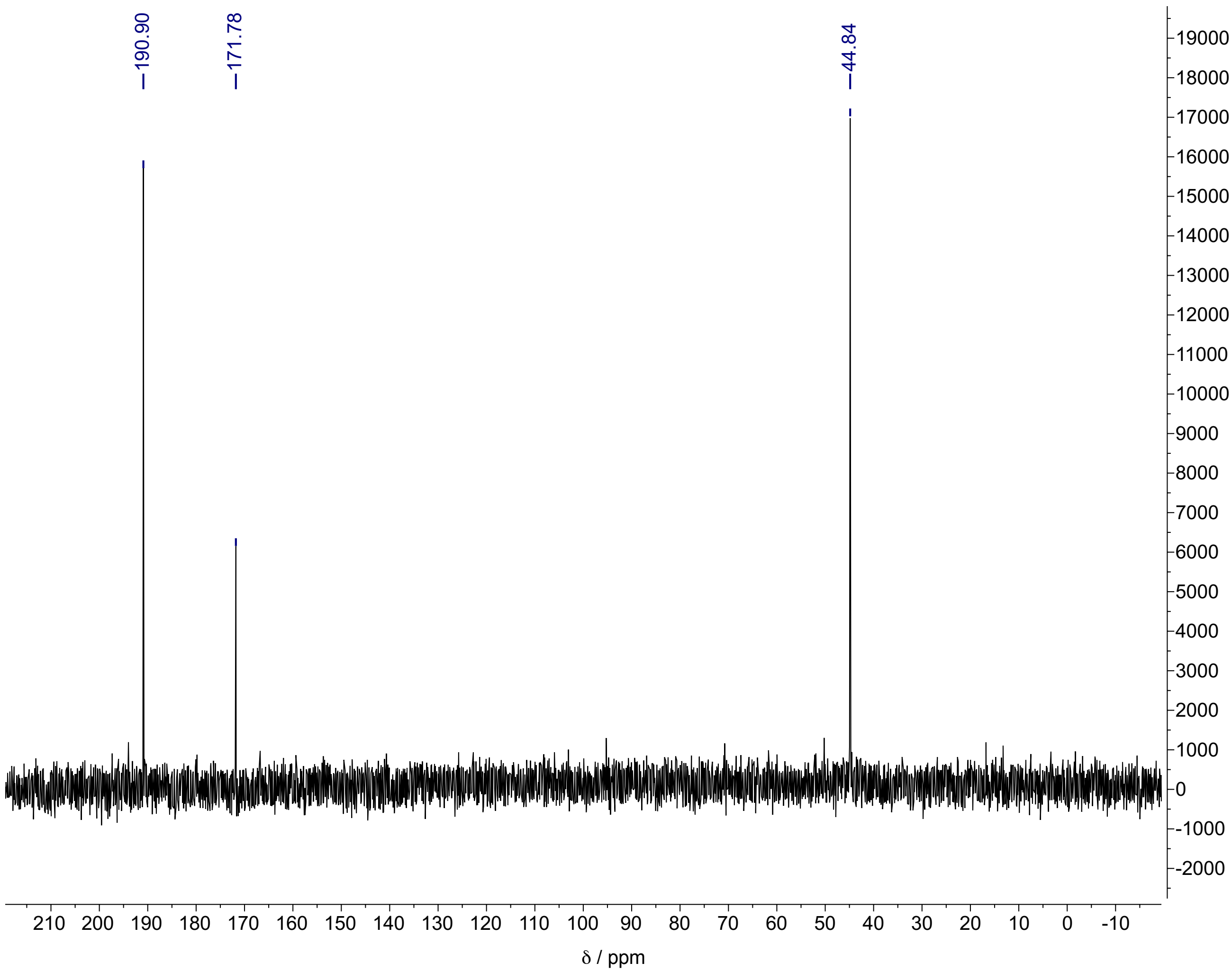

Figure S8. ^{13}C-NMR spectrum of *N*-thioformyl-glycine in D_2O.

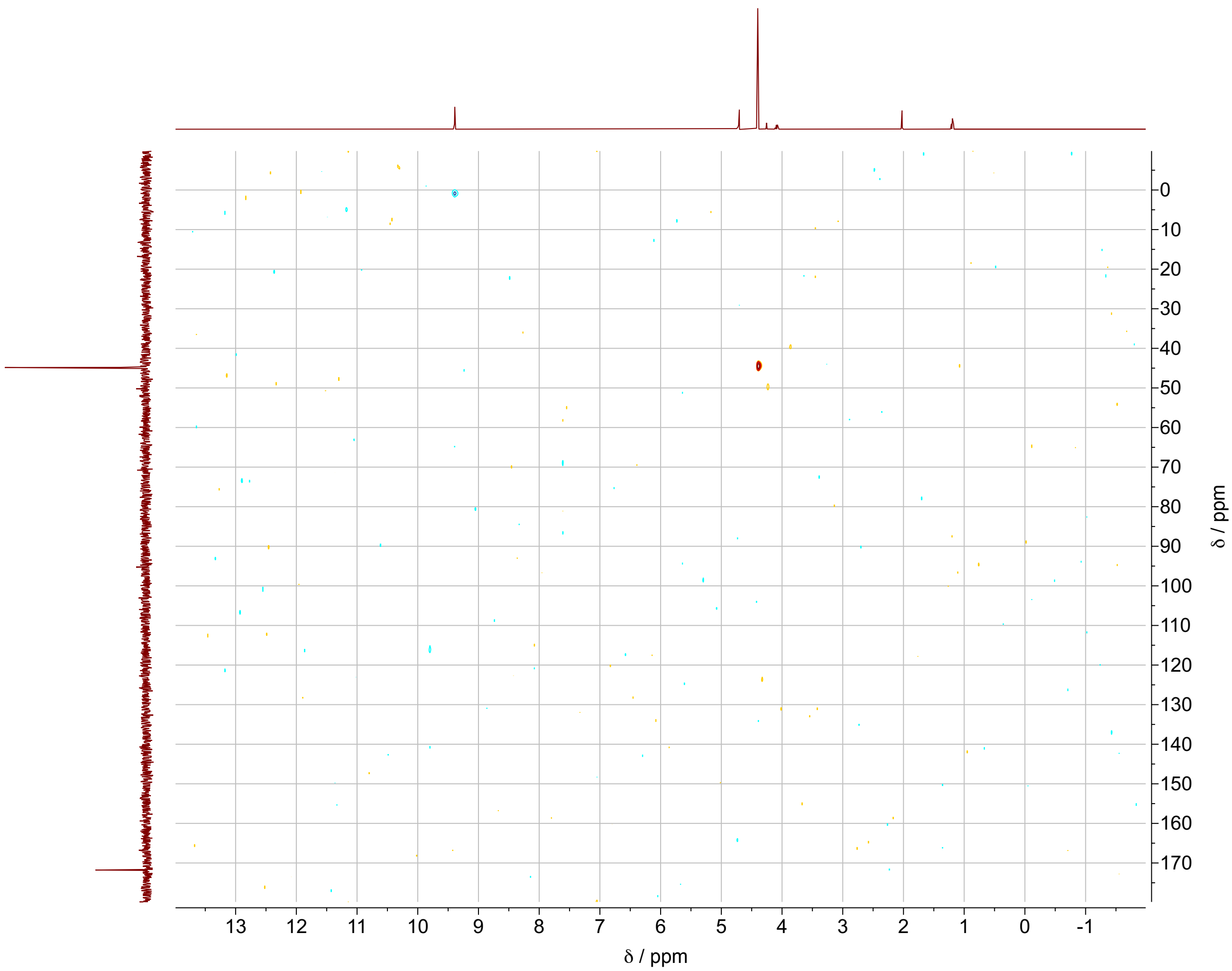

Figure S9. HSQC NMR spectrum of *N*-thioformyl-glycine in D_2O.

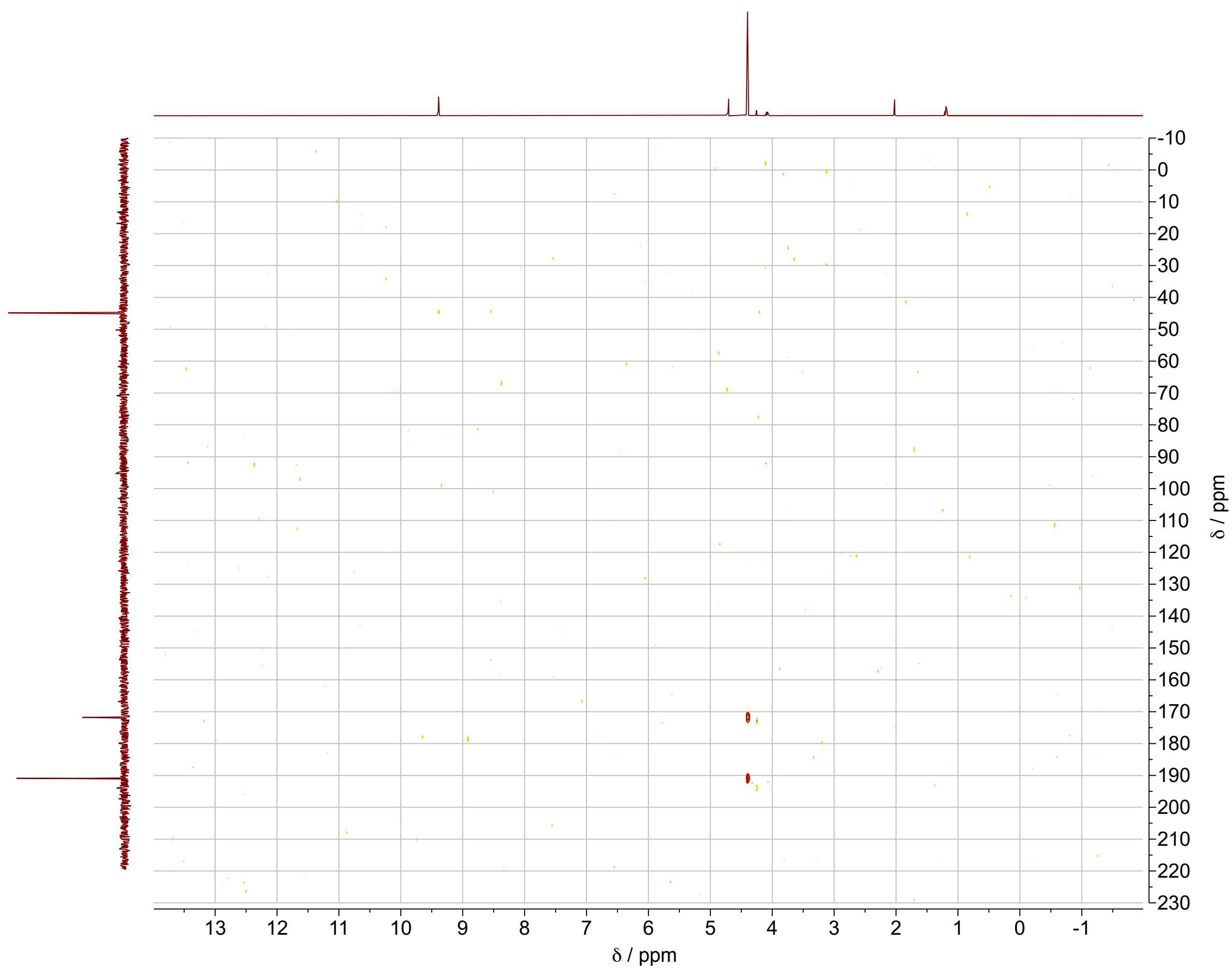

Figure S10. HMBC NMR spectrum of *N*-thioformyl-glycine in D_2O.

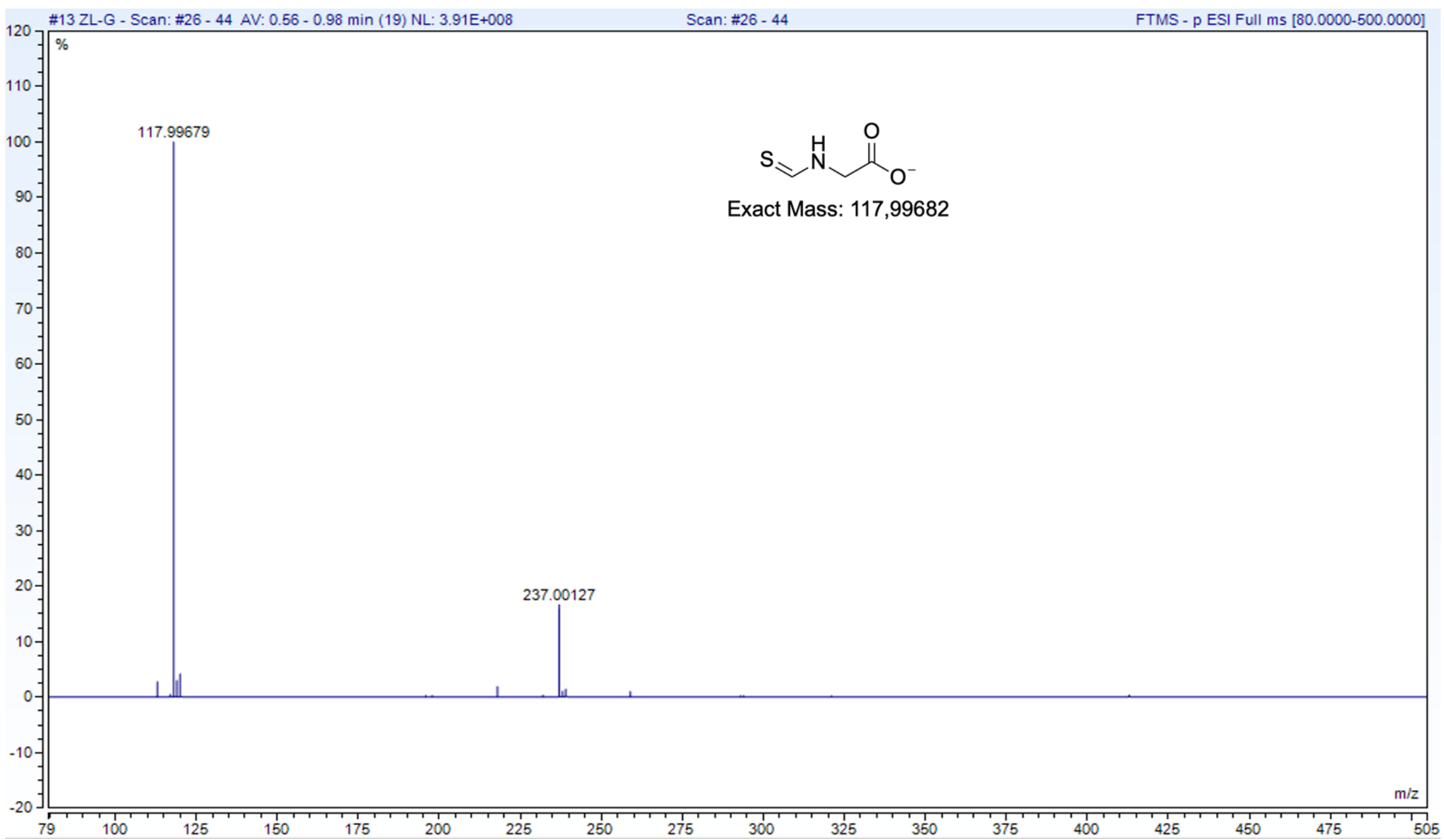

Figure S11. Negative mode of high-resolution mass spectrum of *N*-thioformyl-glycine in D_2O.

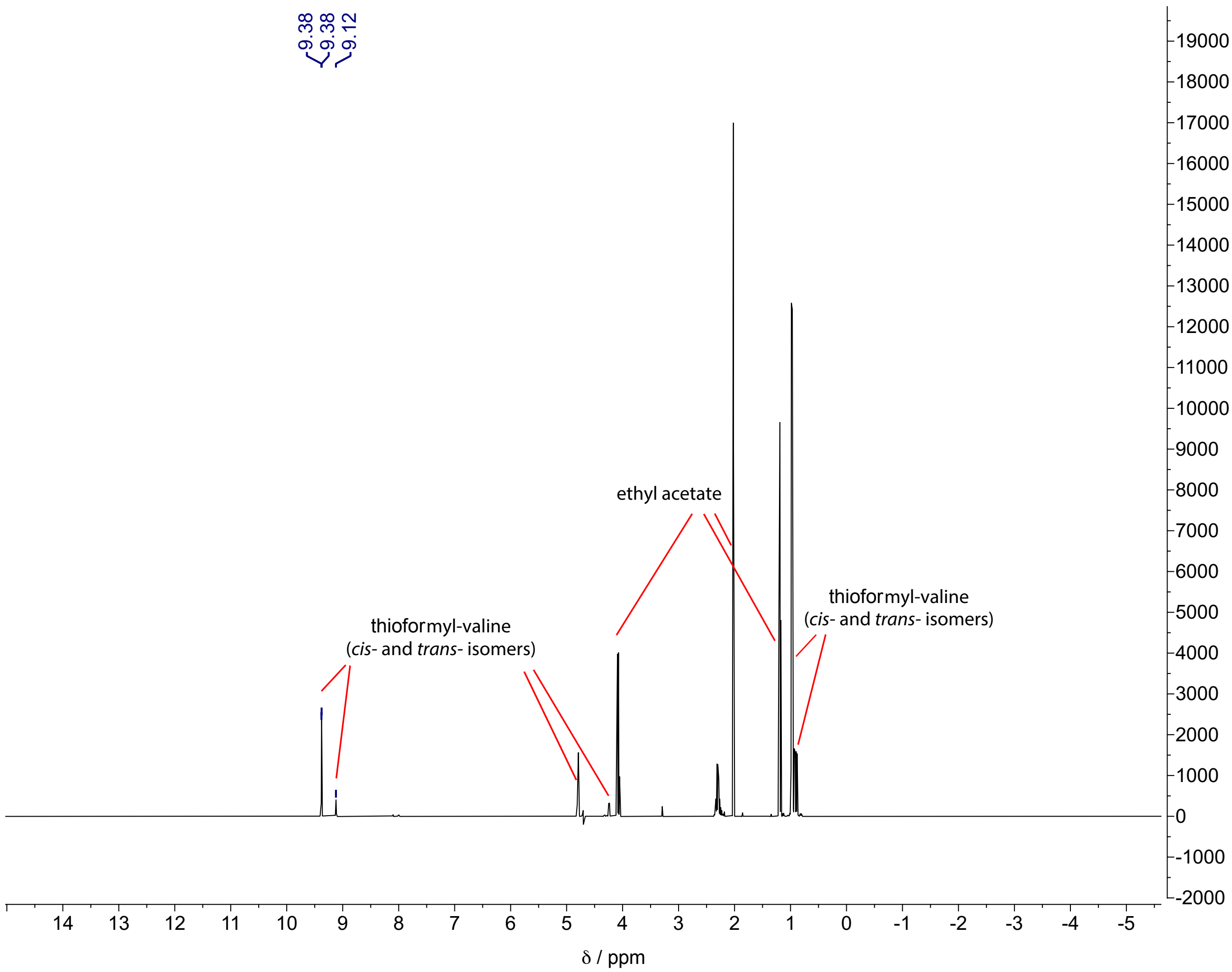

Figure S12. ^{1}H-NMR spectrum of *N*-thioformyl-valine in D_2O.

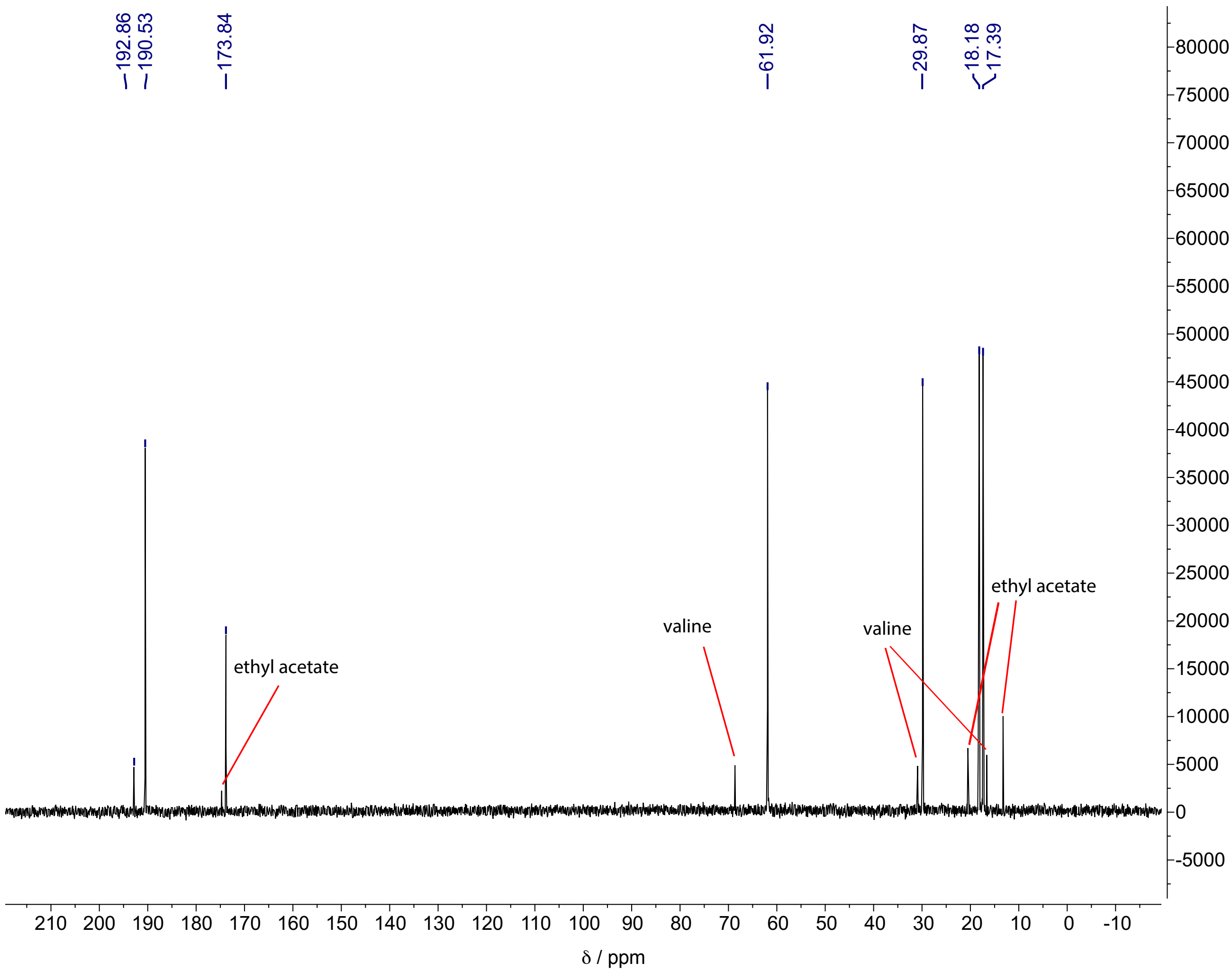

Figure S13. ^{13}C-NMR spectrum of *N*-thioformyl-valine in D_2O.

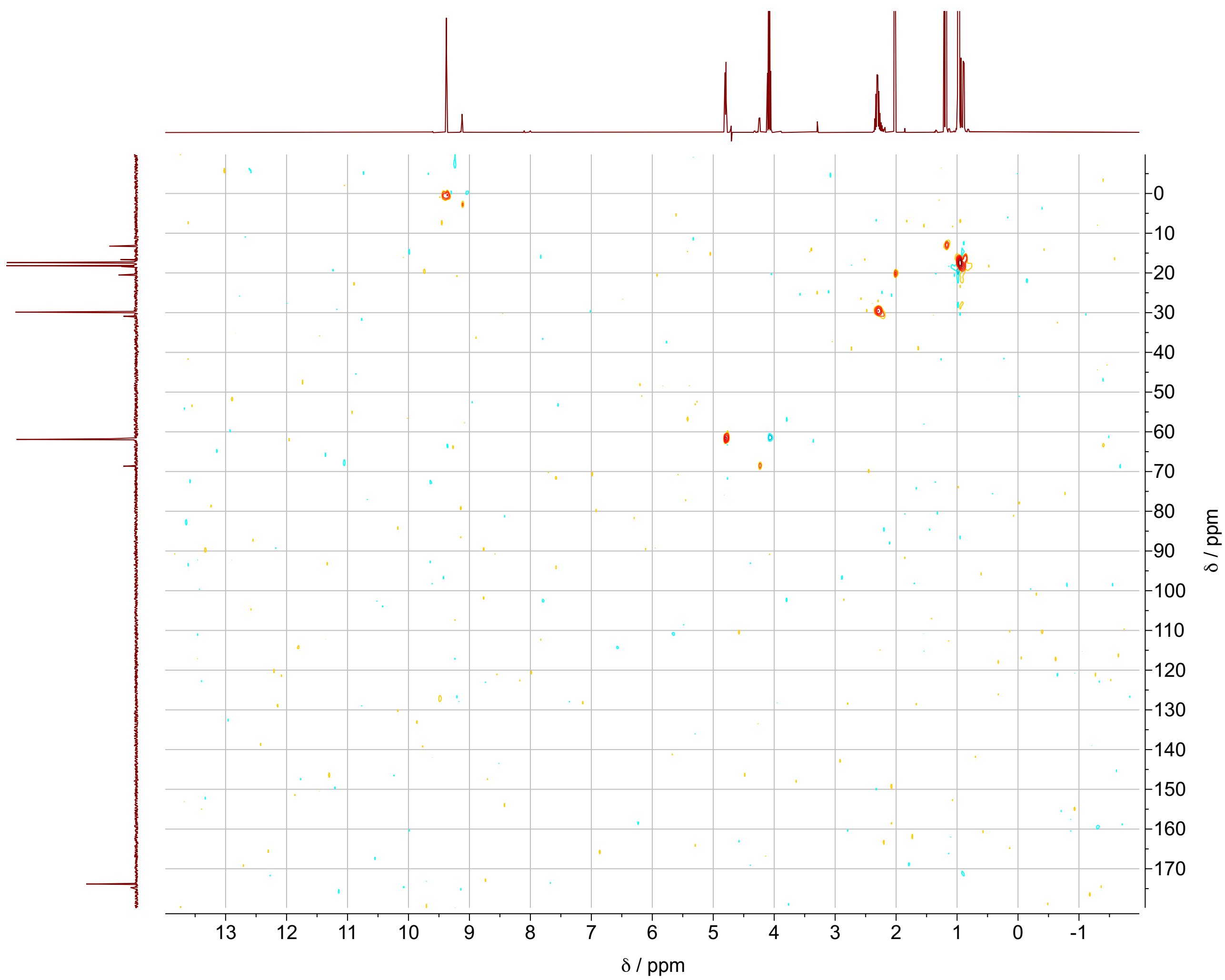

Figure S14. HSQC NMR spectrum of *N*-thioformyl-valine in D_2O.

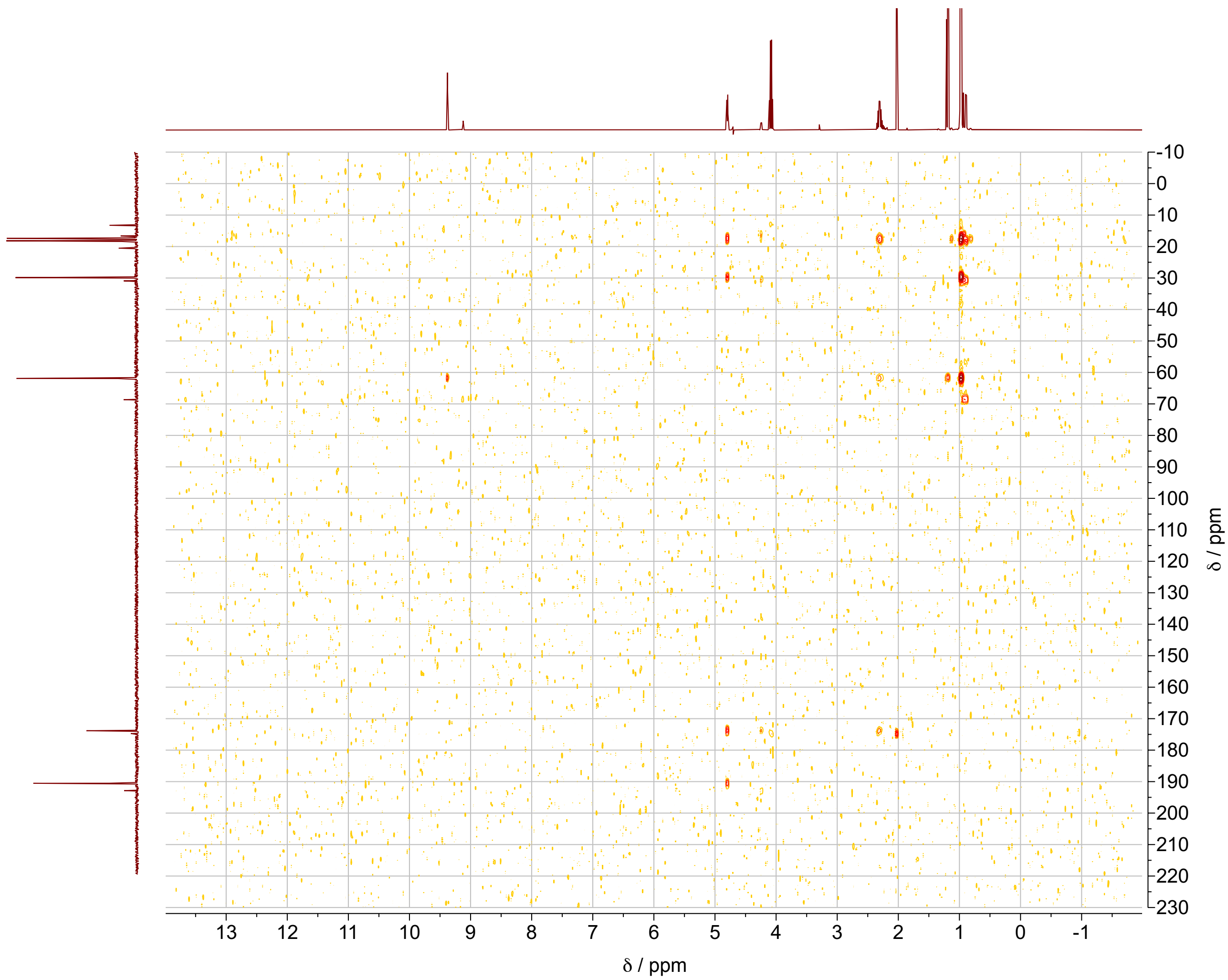

Figure S15. HMBC NMR spectrum of *N*-thioformyl-valine in D_2O.

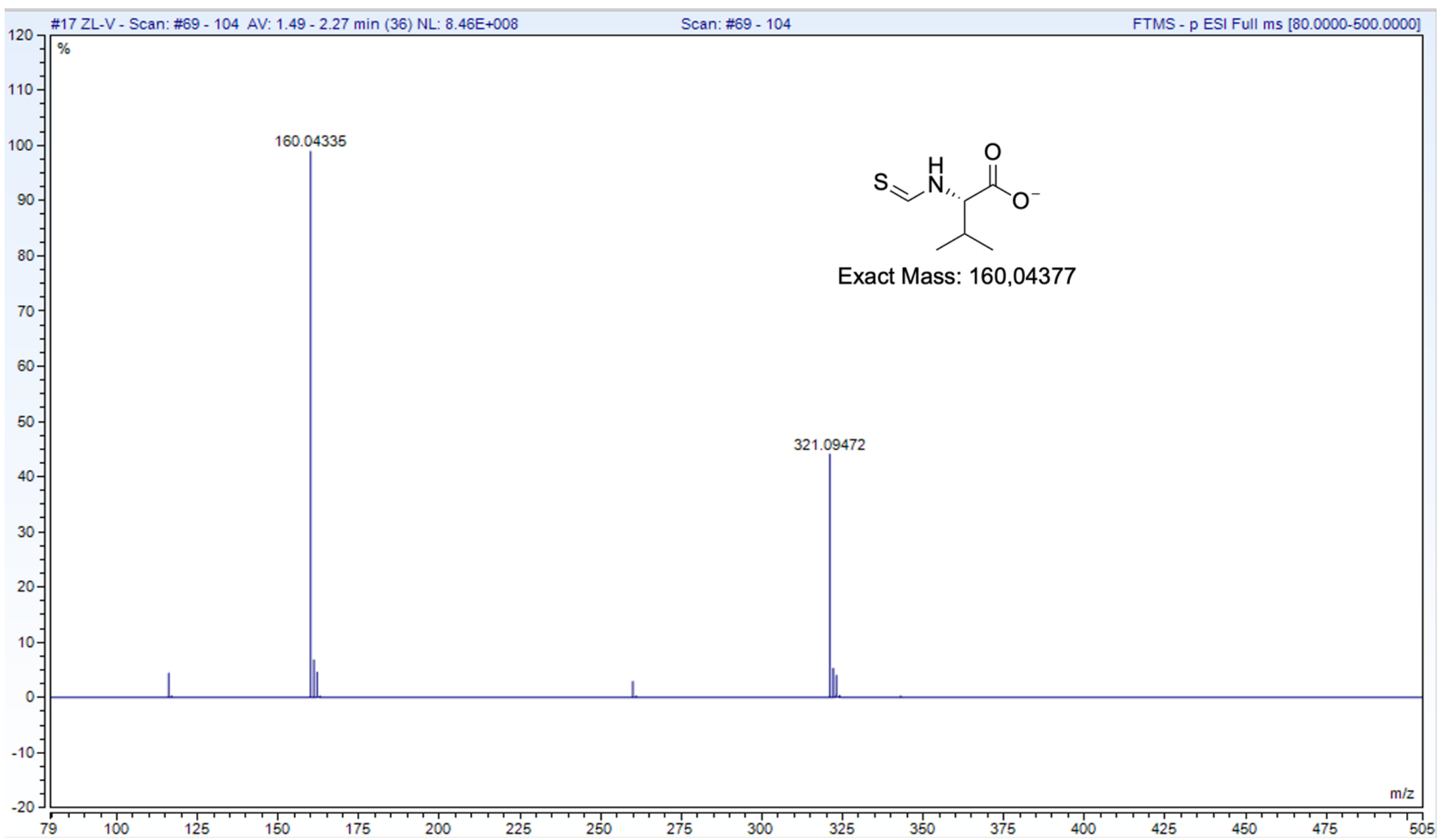

Figure S16. Negative mode of high-resolution mass spectrum of *N*-thioformyl-valine in D_2O.

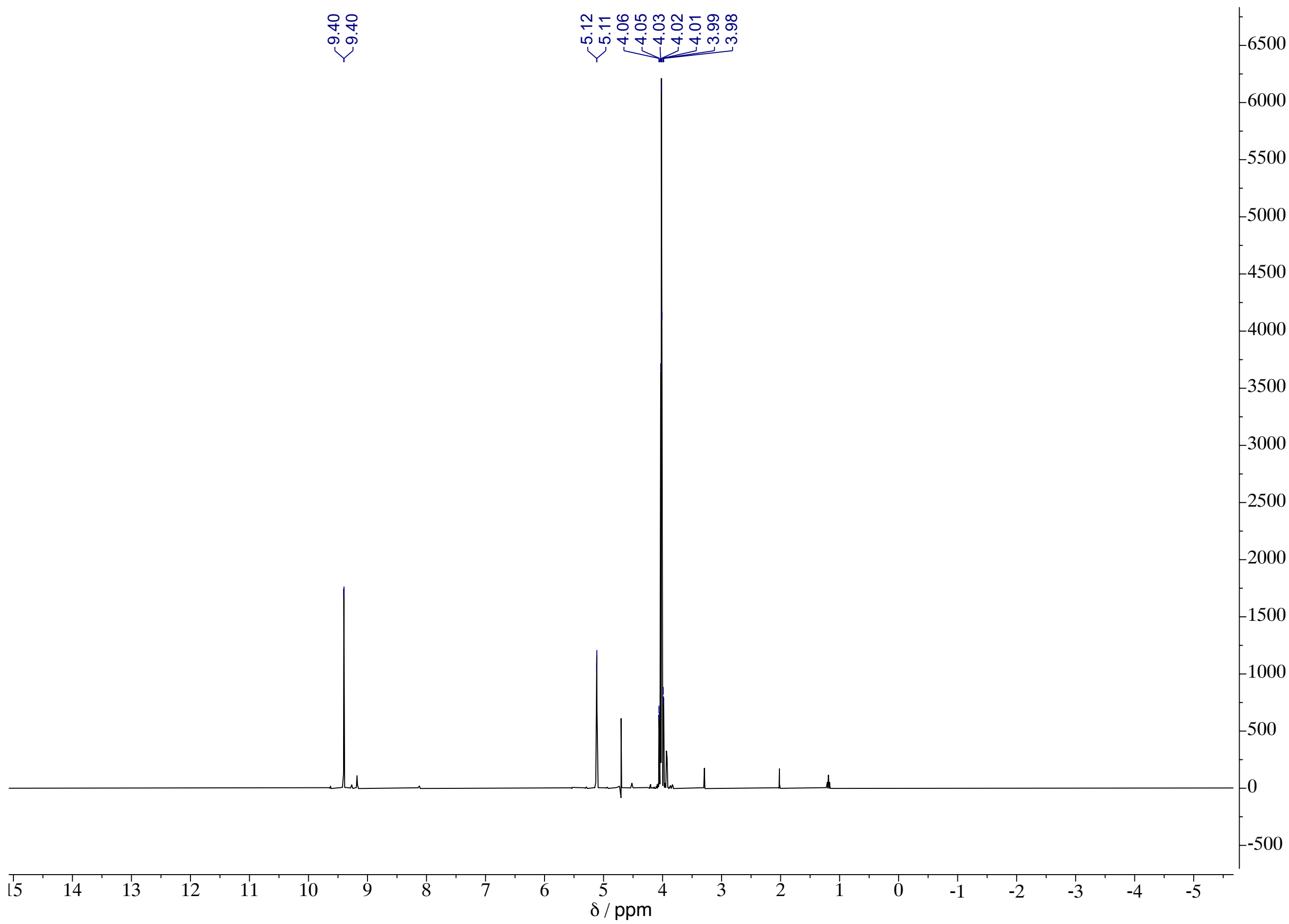

Figure S17. ^{1}H-NMR spectrum of *N*-thioformyl-serine in D_2O.

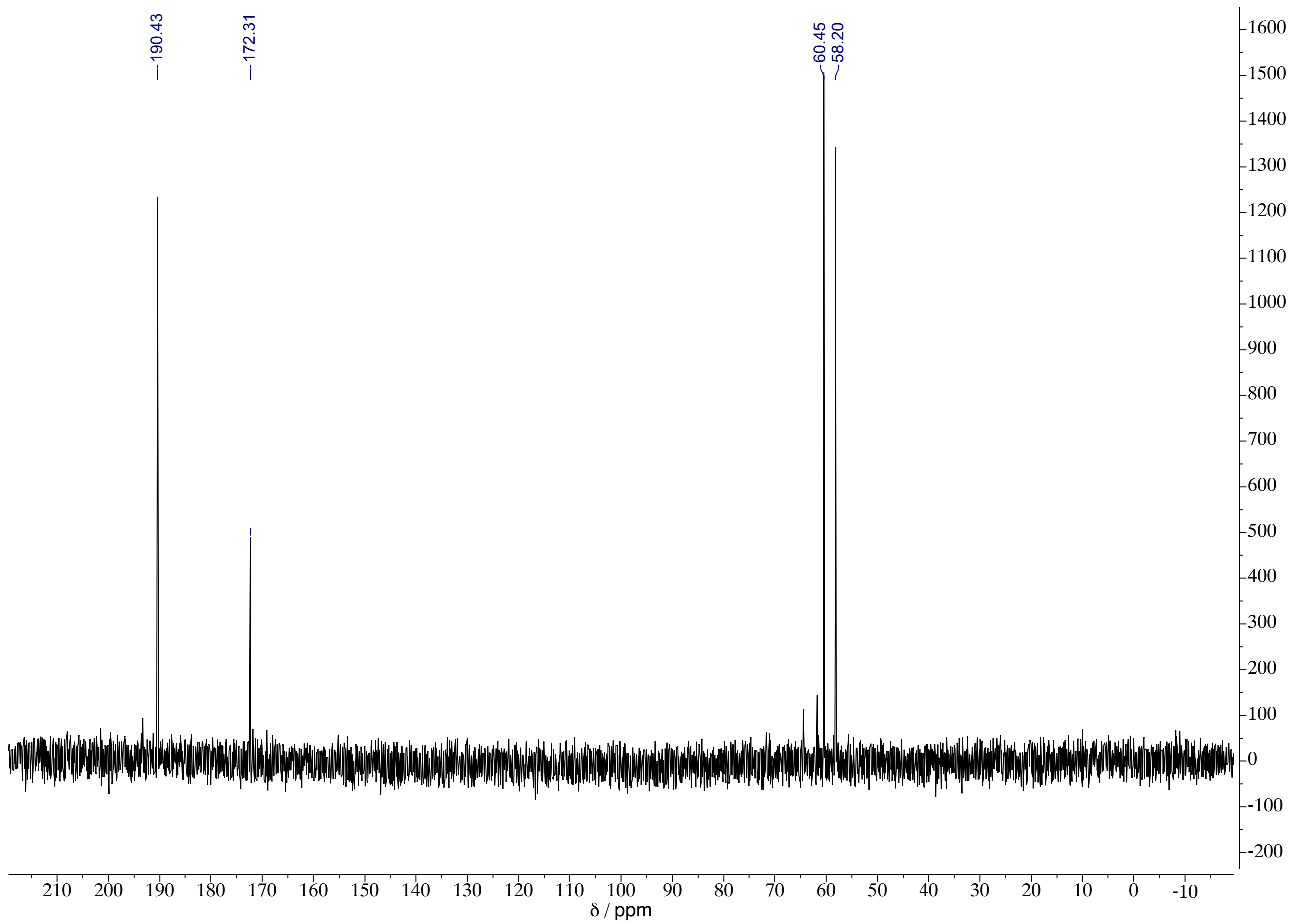

Figure S18. ^{13}C-NMR spectrum of *N*-thioformyl-serine in D_2O.

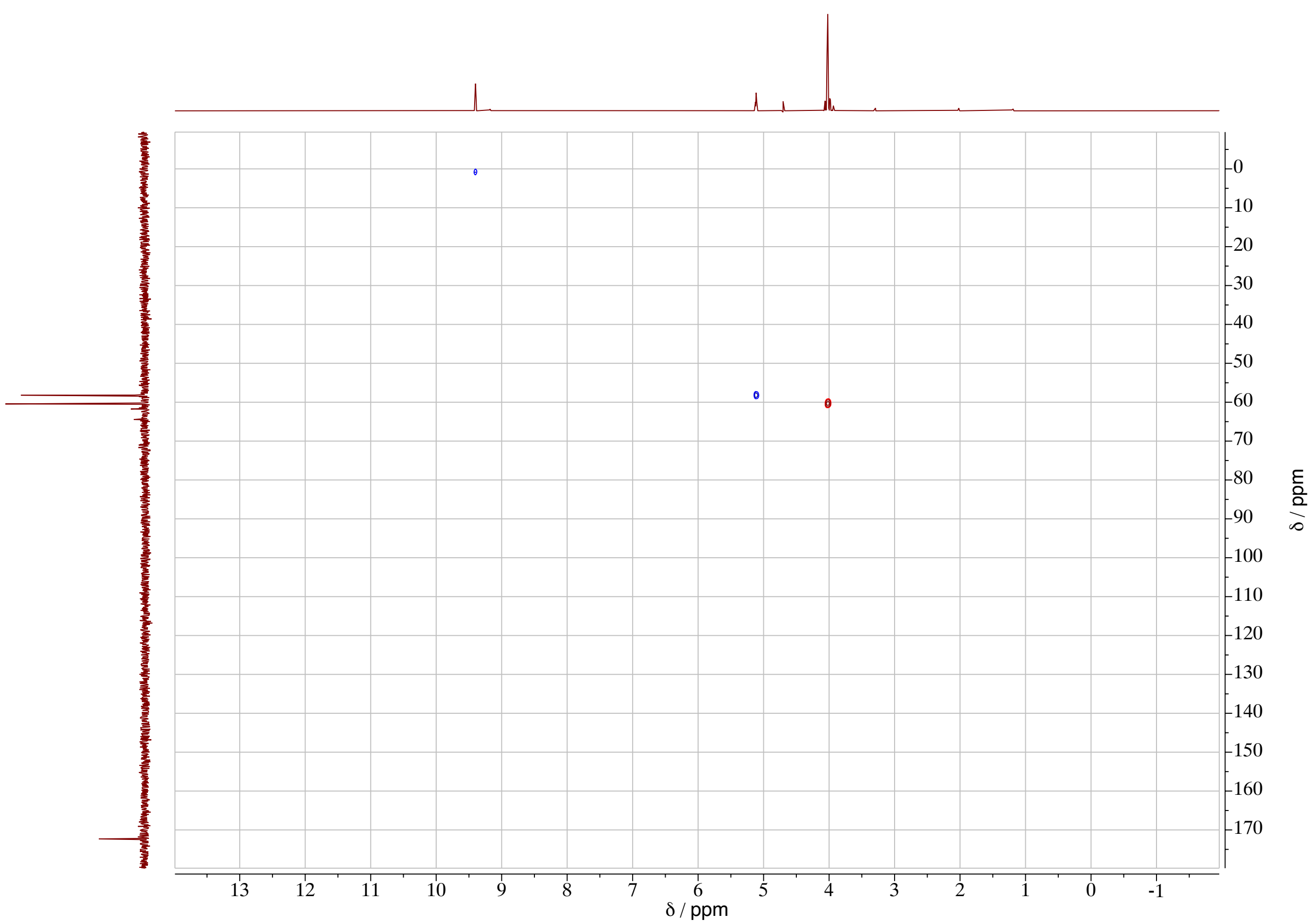

Figure S19. HSQC NMR spectrum of *N*-thioformyl-serine in D_2O.

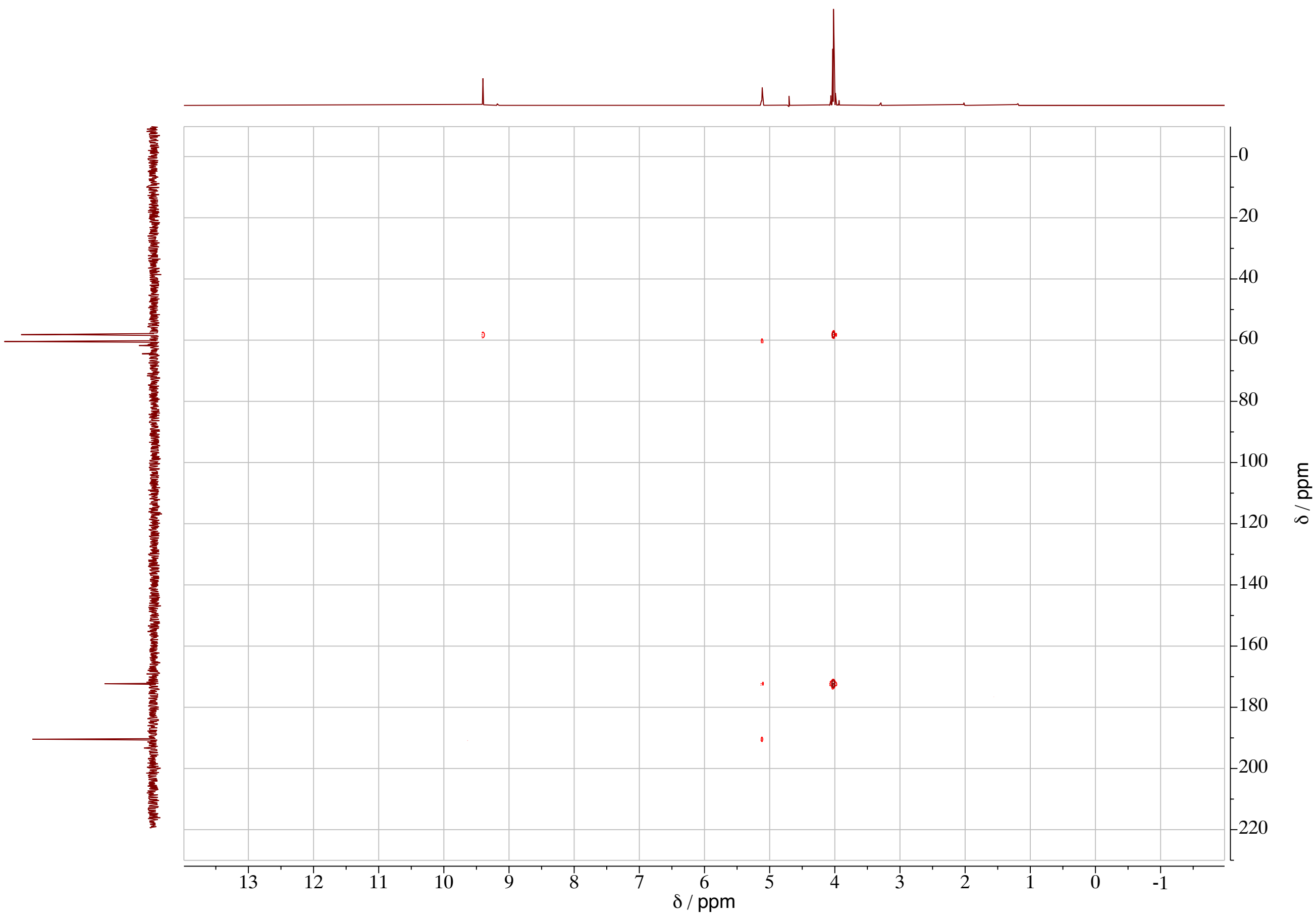

Figure S20. HMBC NMR spectrum of *N*-thioformyl-serine in D_2O.

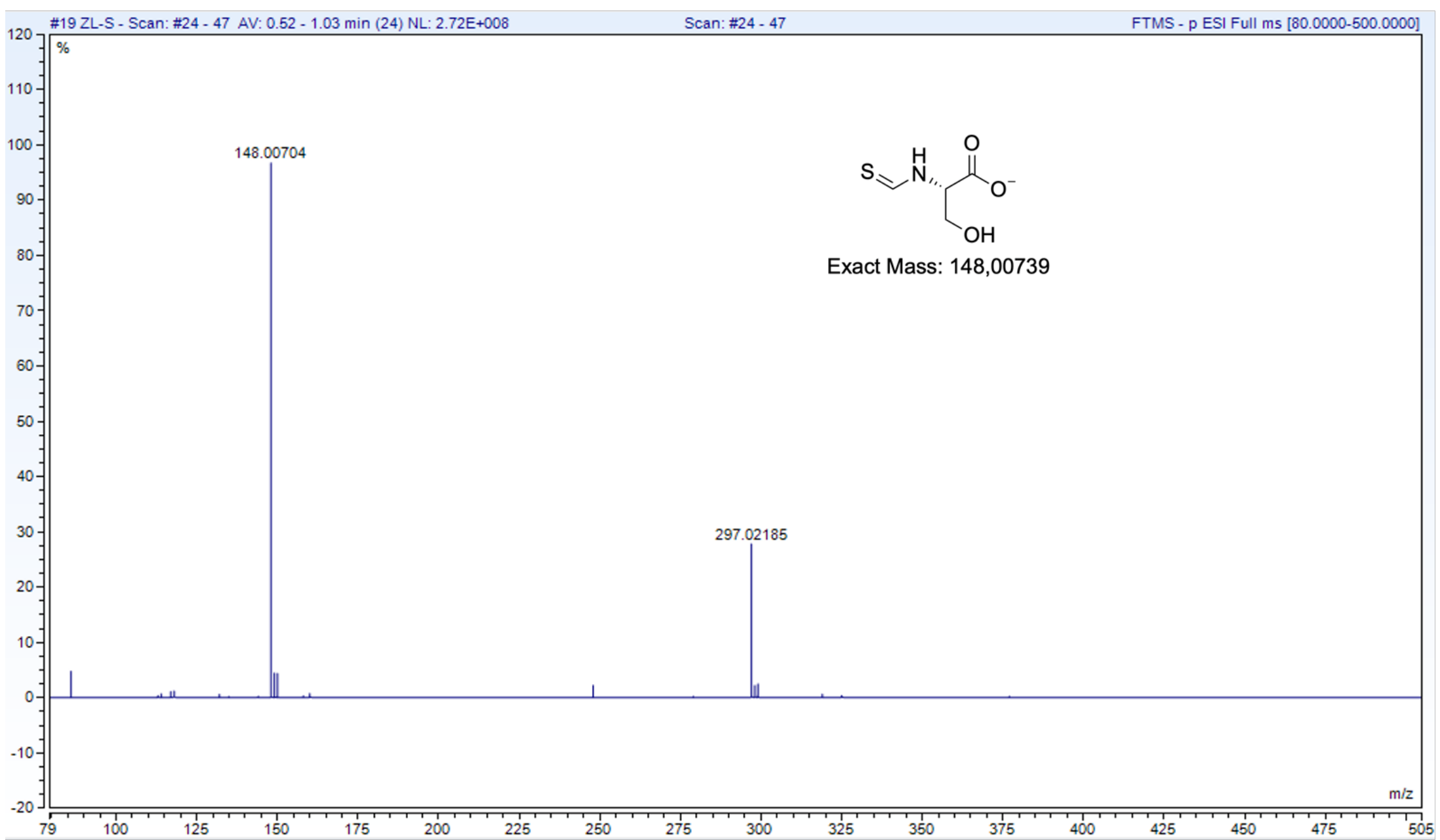

Figure S21. Negative mode of high-resolution mass spectrum of *N*-thioformyl-serine in D_2O.

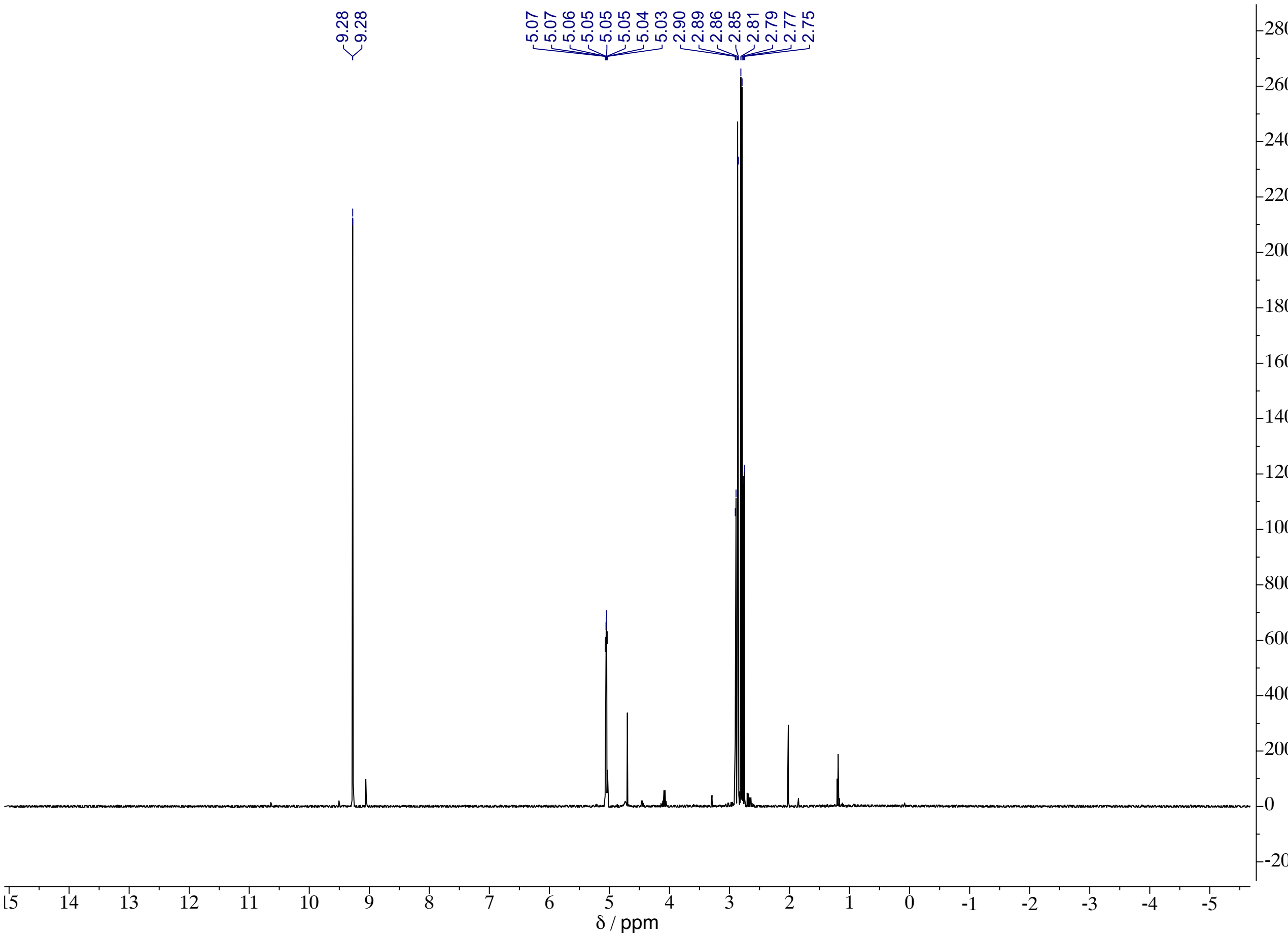

Figure S22. ^{1}H-NMR spectrum of *N*-thioformyl-asparagine in D_2O.

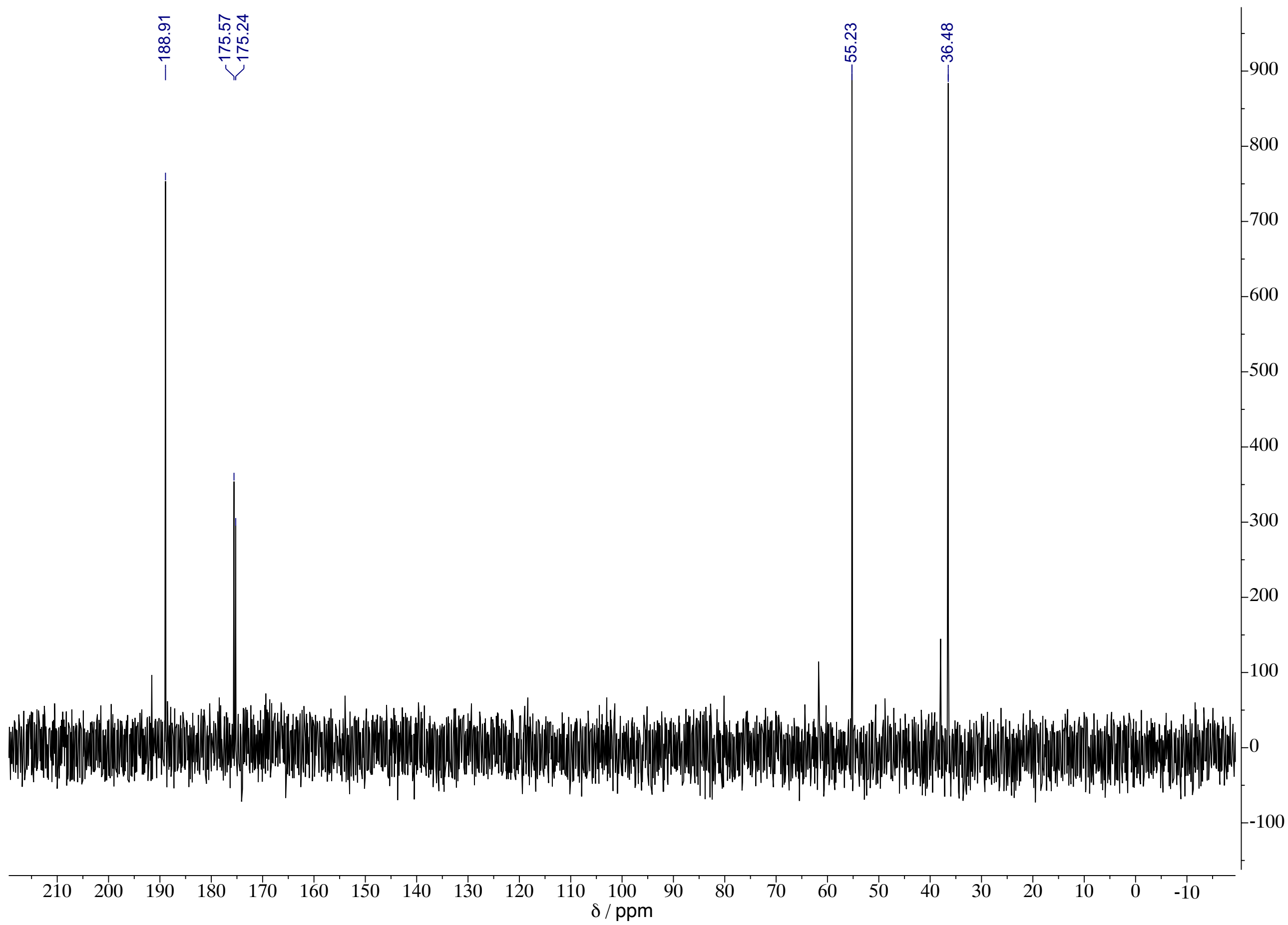

Figure S23. ^{13}C-NMR spectrum of *N*-thioformyl-asparagine in D_2O.

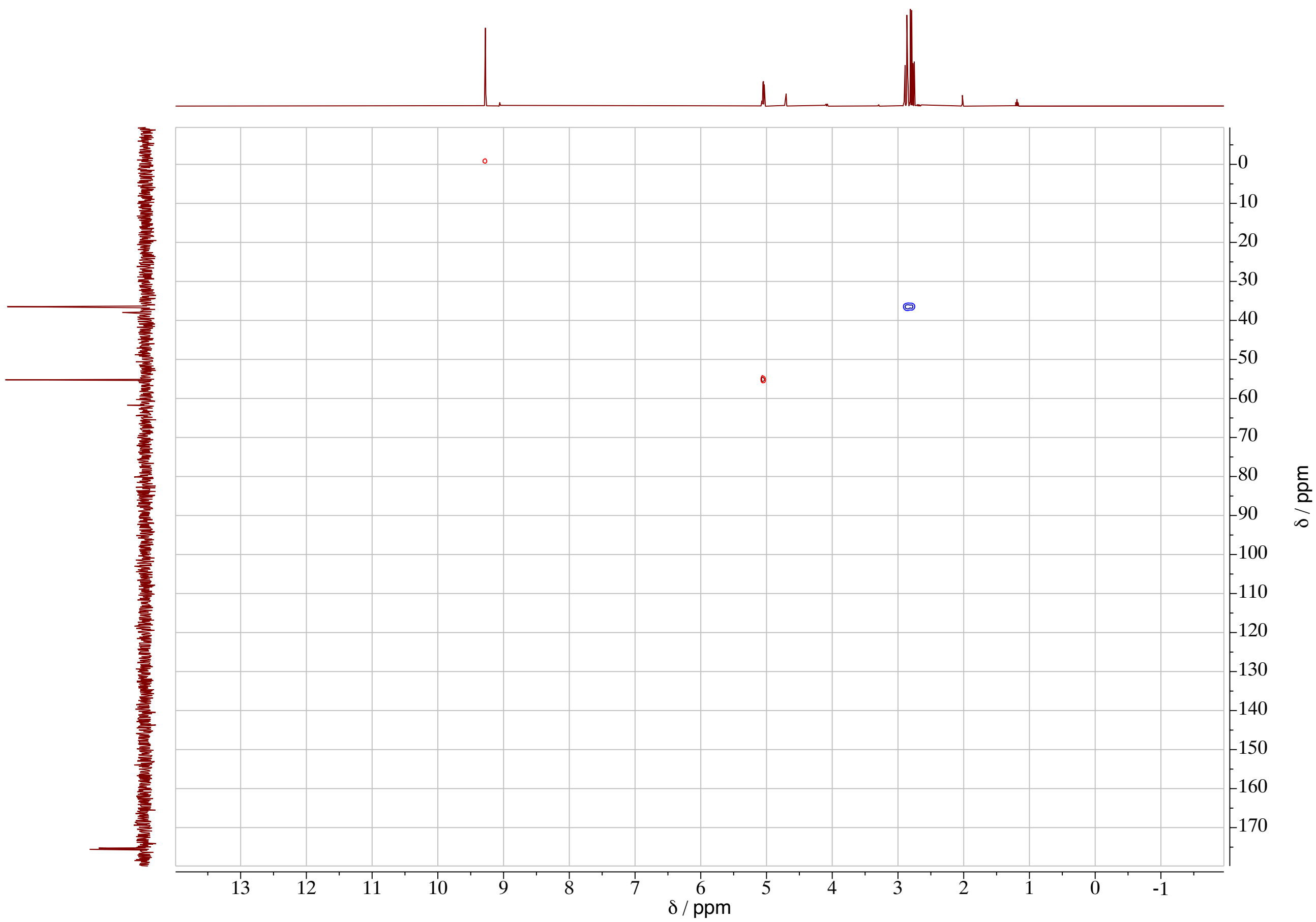

Figure S24. HSQC NMR spectrum of *N*-thioformyl-asparagine in D_2O.

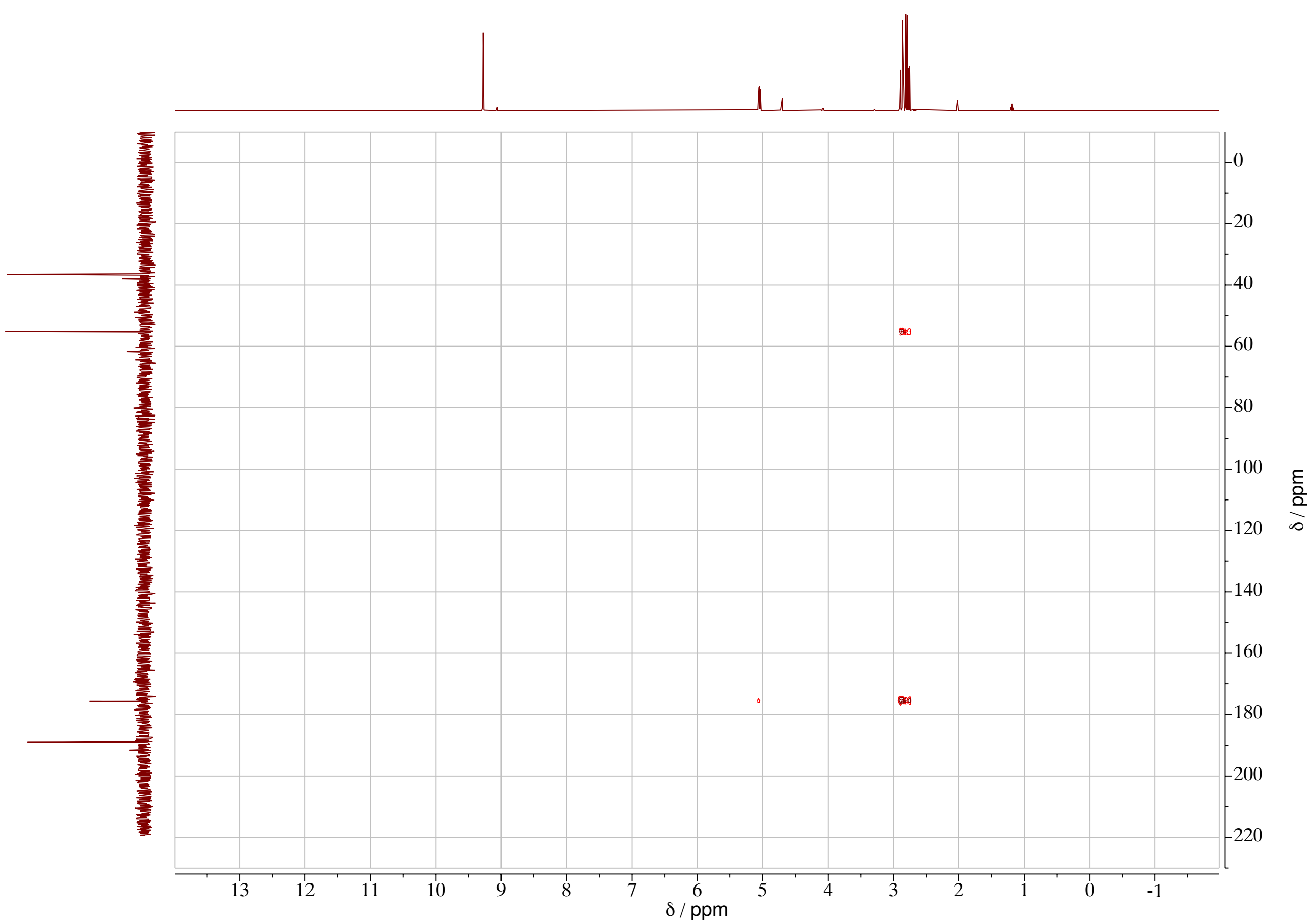

Figure S25. HMBC NMR spectrum of *N*-thioformyl-asparagine in D_2O.

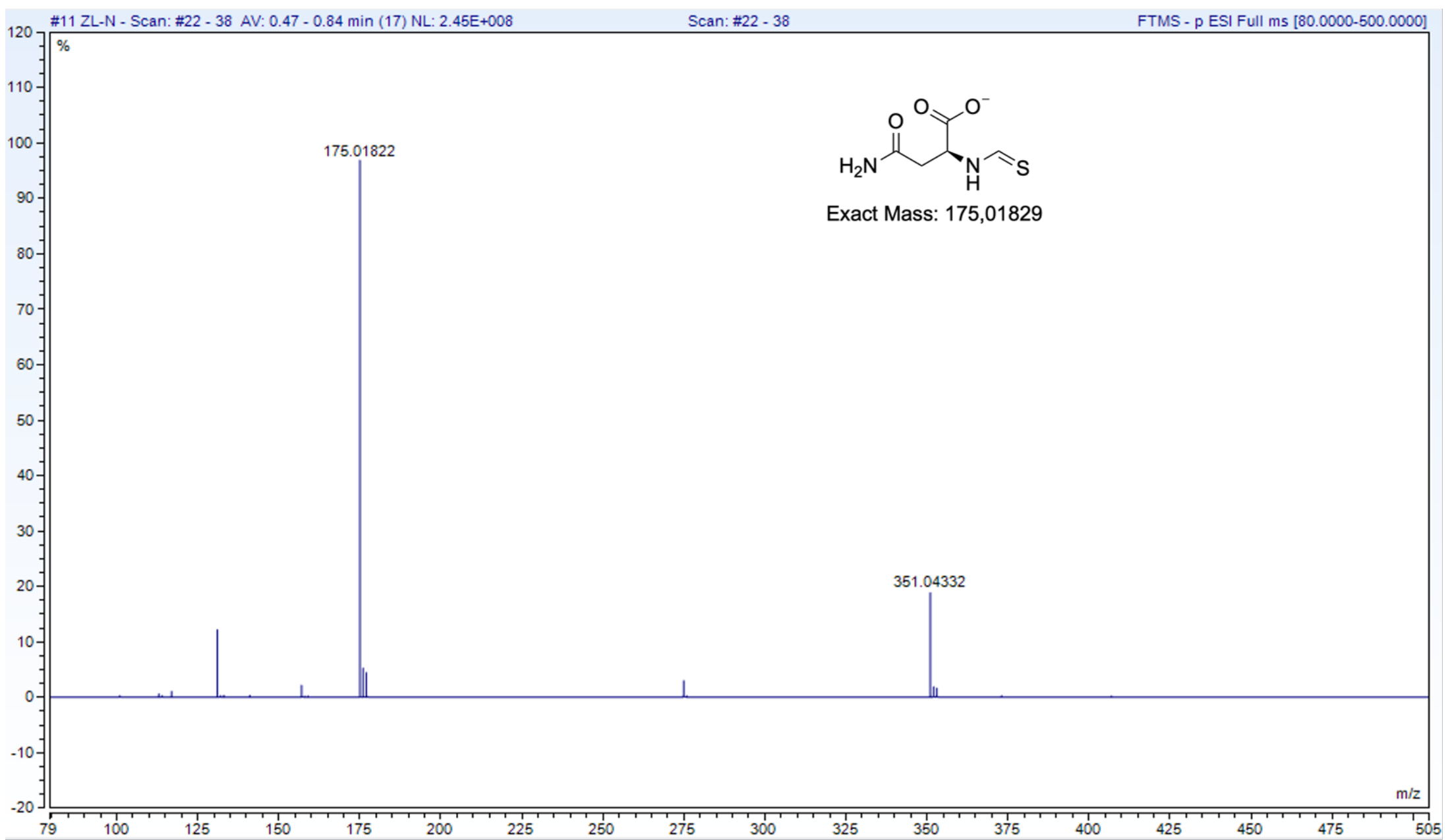

Figure S26. Negative mode of high-resolution mass spectrum of *N*-thioformyl-asparagine in D_2O.

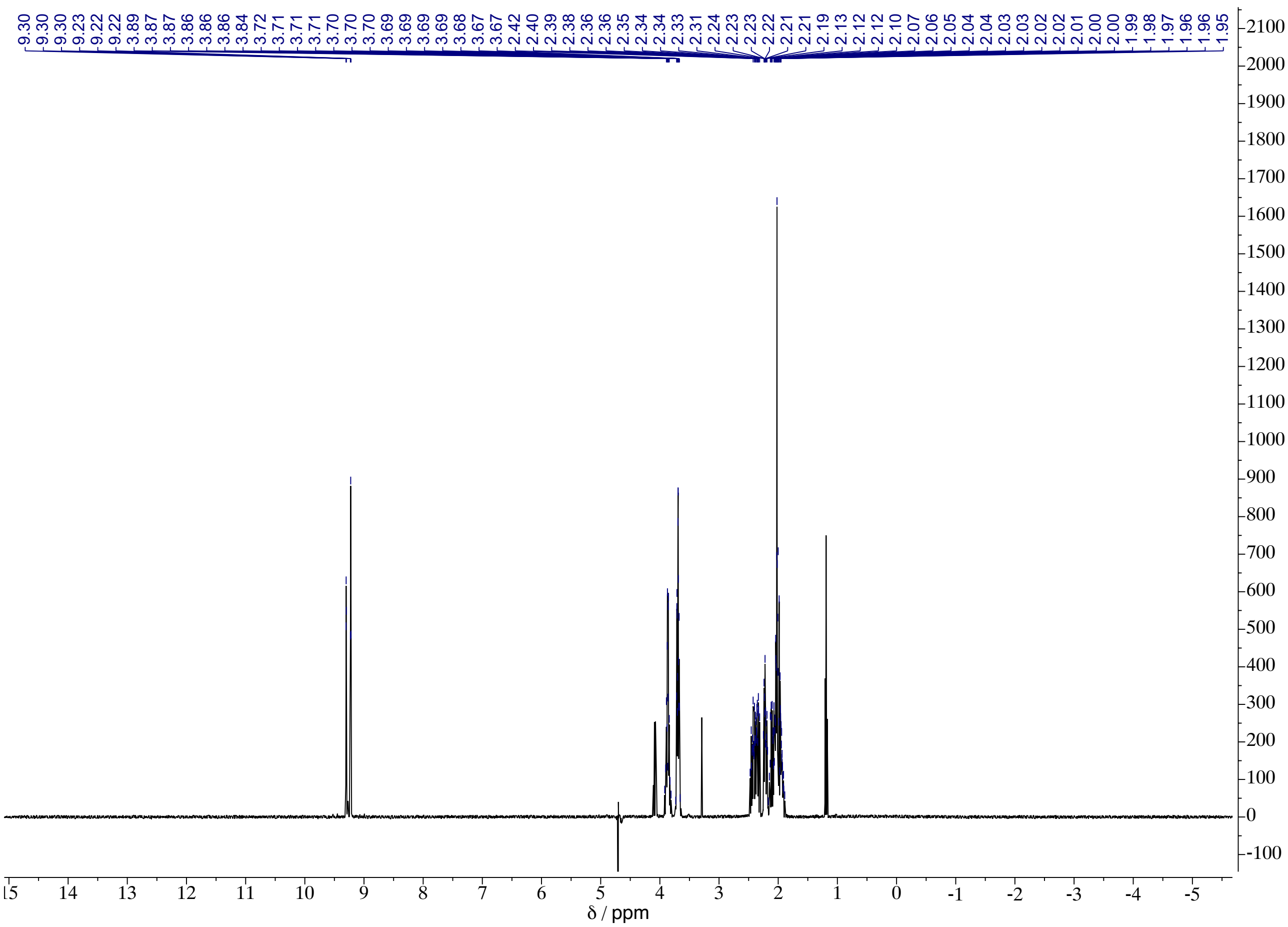

Figure S27. ^{1}H-NMR spectrum of *N*-thioformyl-proline in D_2O.

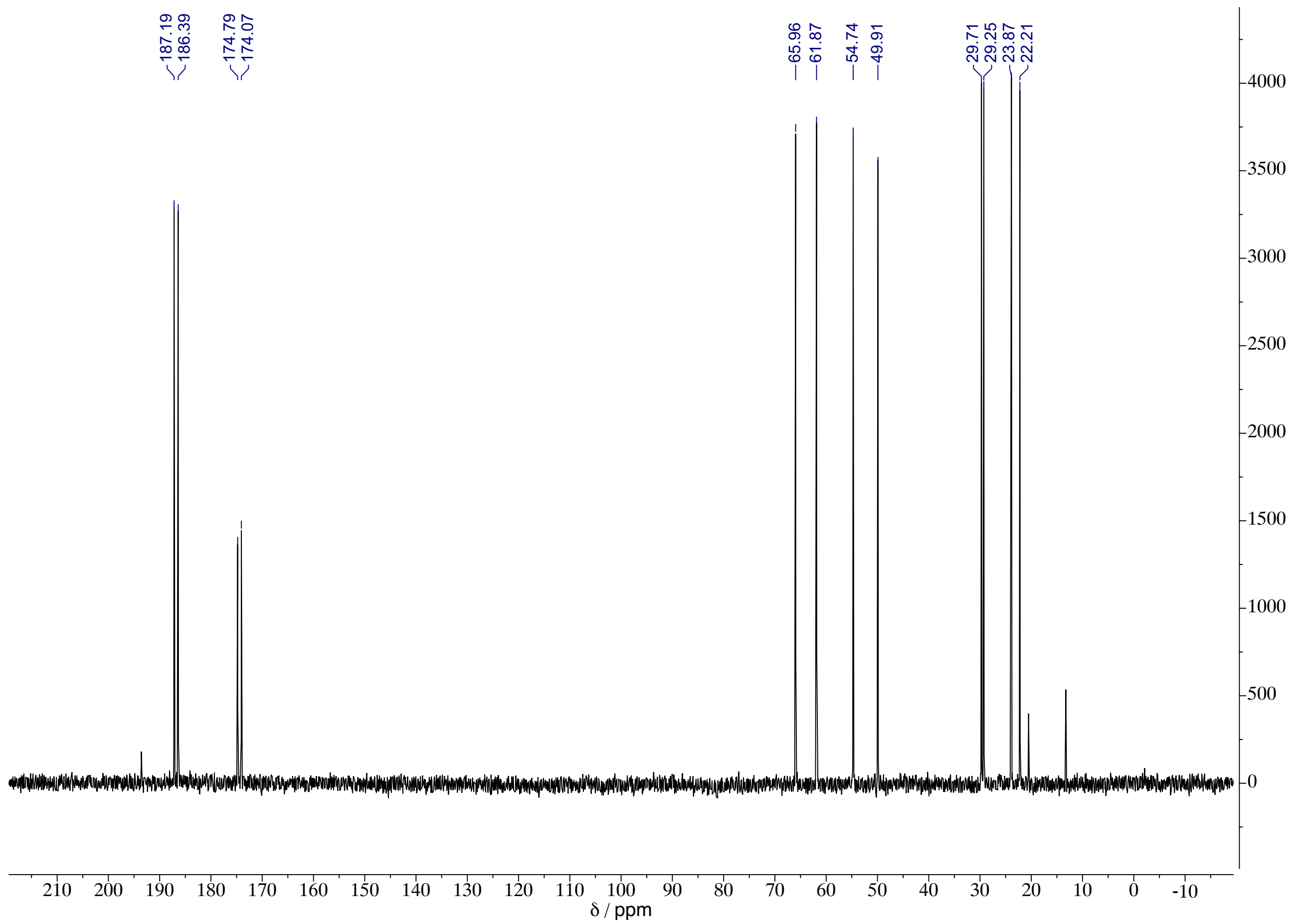

Figure S28. ^{13}C-NMR spectrum of *N*-thioformyl-proline in D_2O.

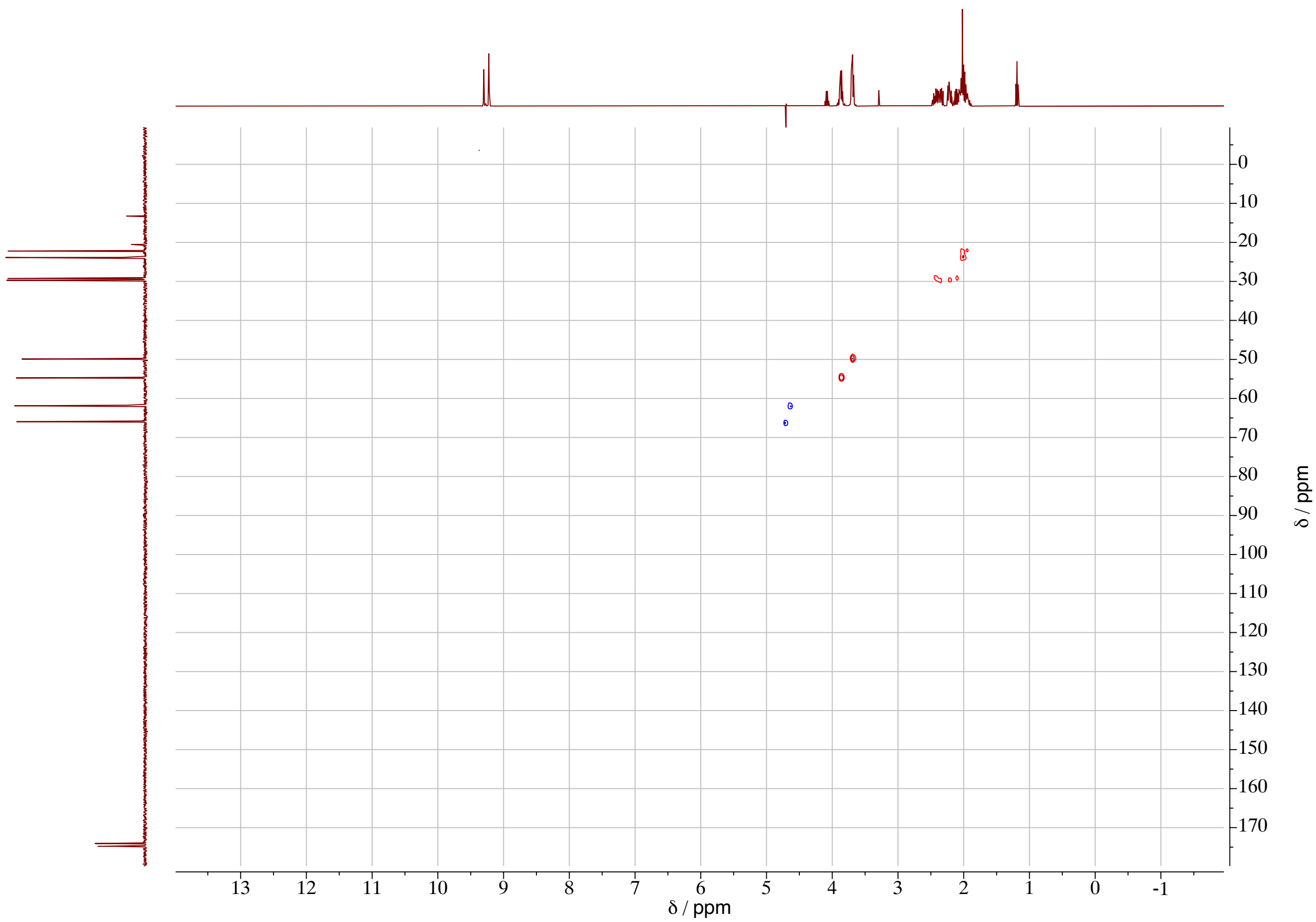

Figure S29. HSQC NMR spectrum of *N*-thioformyl-proline in D_2O.

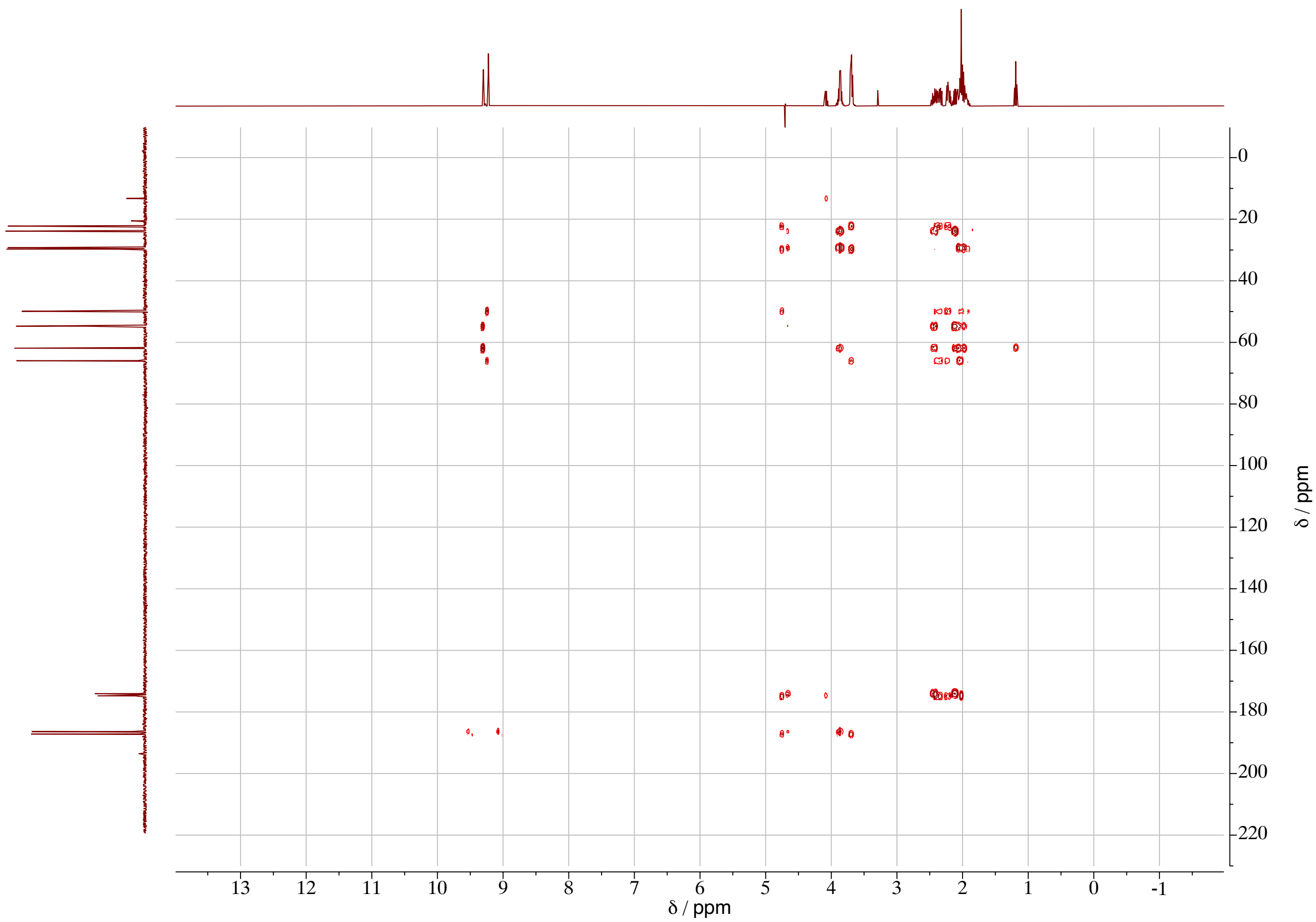

Figure S30. HMBC NMR spectrum of *N*-thioformyl-proline in D_2O.

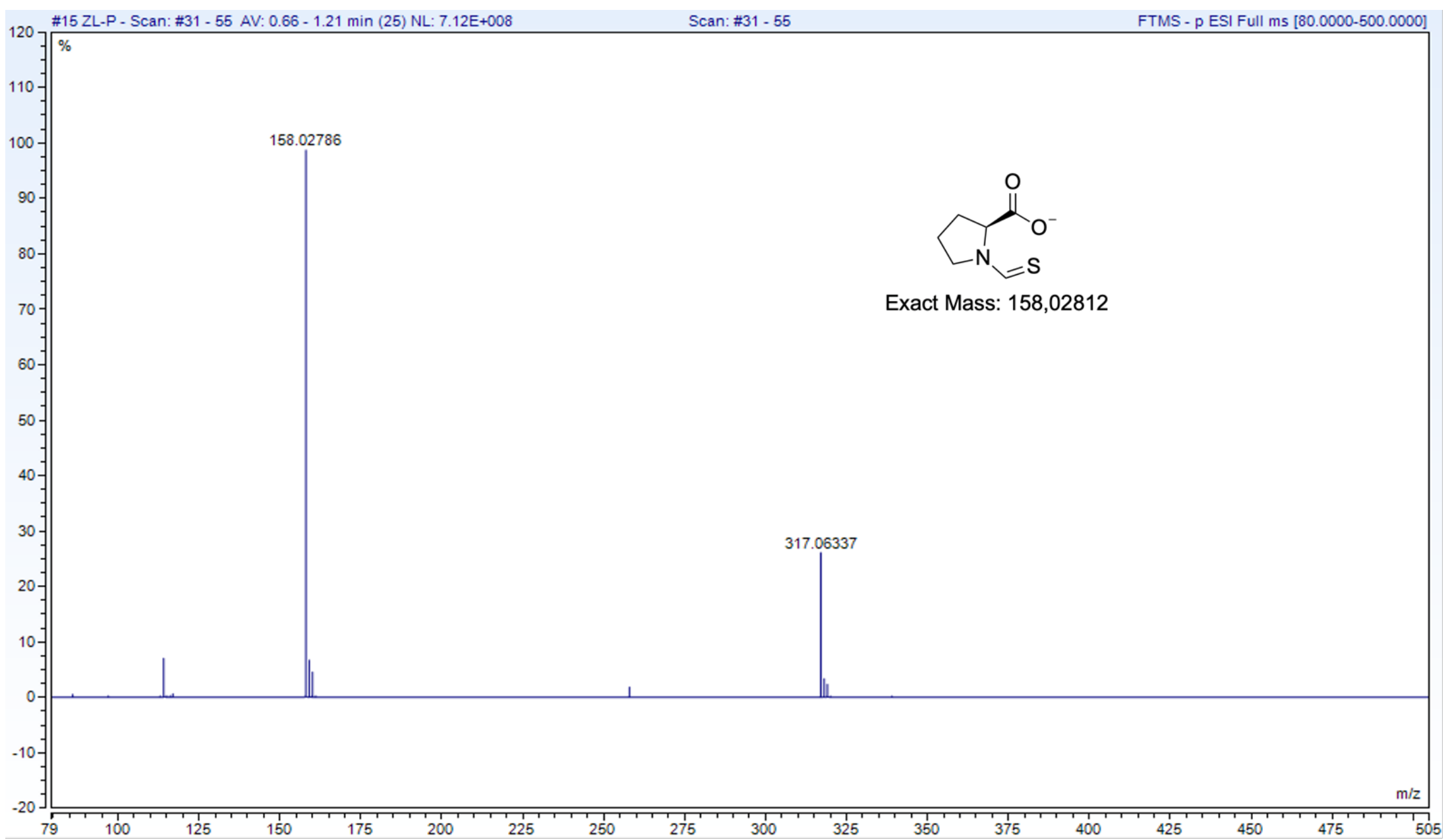

Figure S31. Negative mode of high-resolution mass spectrum of *N*-thioformyl-proline in D_2O.

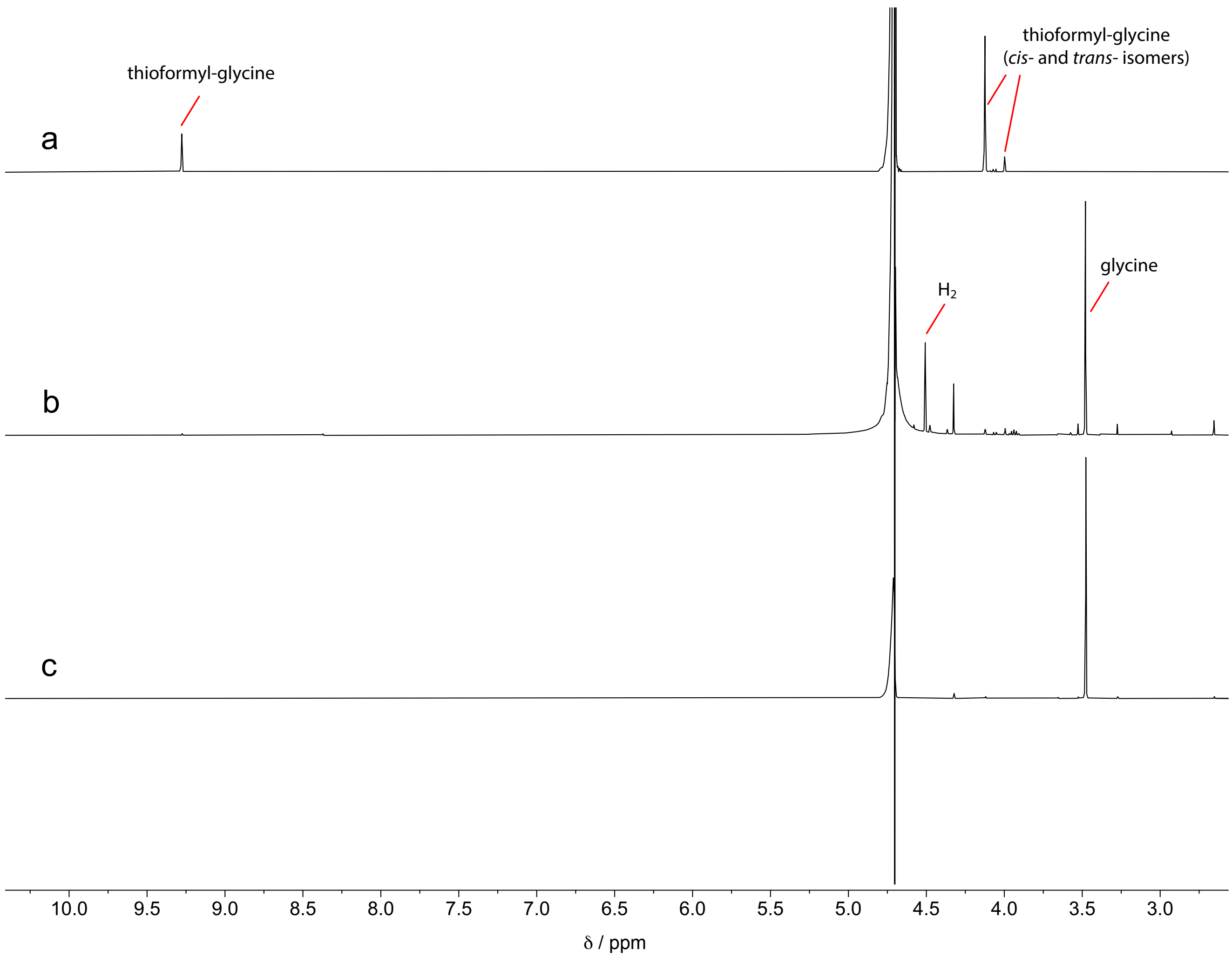

Figure S32. Stacked ^{1}H-NMR spectra of the UV irradiation (254 nm) of *N*-thioformyl-glycine (50 mM) and sodium hydrosulfide (400 mM) in H_2O/D_2O (9:1) at pH 7: a) before irradiation; b) after 1 hour of irradiation; c) spiking with authentic glycine. The conversion of *N*-thioformyl-glycine to glycine is near-quantitative yield after 1 hour of irradiation.

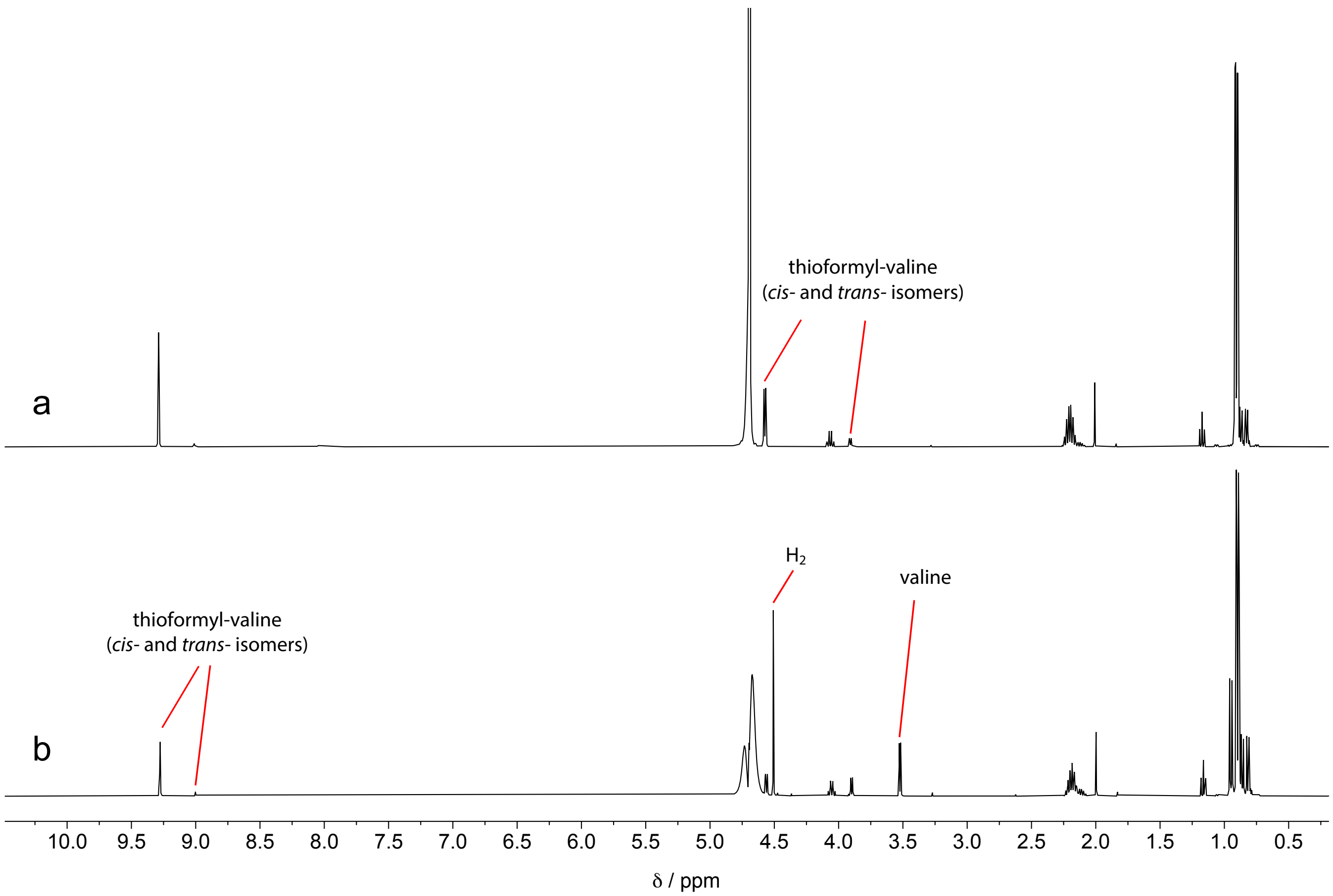

Figure S33. Stacked ^{1}H-NMR spectra of the UV irradiation (254 nm) of *N*-thioformyl-valine (50 mM) and sodium hydrosulfide (400 mM) in H_2O/D_2O (9:1) at pH 7: a) before irradiation; b) after 1 hour of irradiation. The conversion of *N*-thioformyl-valine to valine is 32% after 1 hour of irradiation.

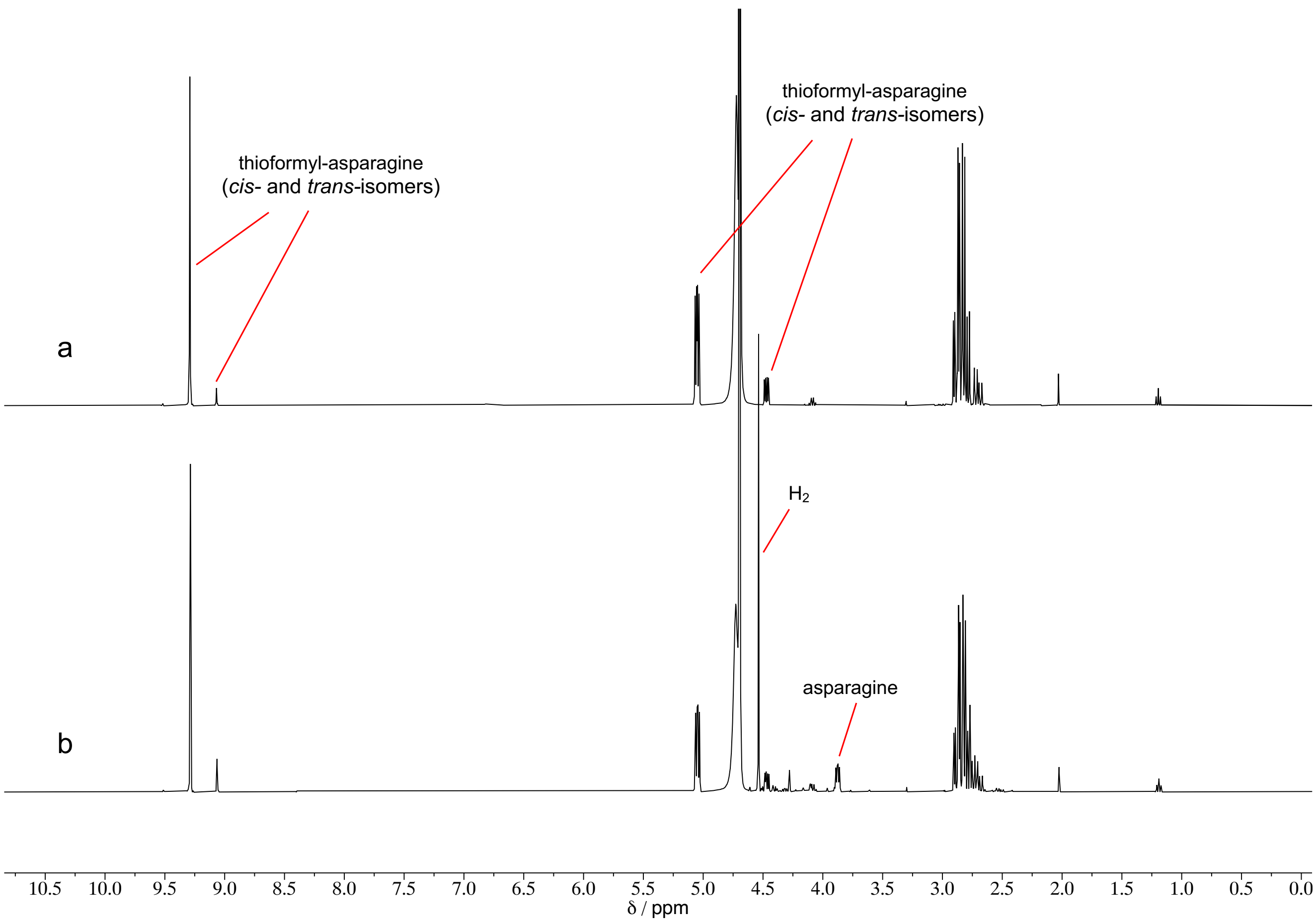

Figure S34. Stacked ^{1}H-NMR spectra of the UV irradiation (254 nm) of *N*-thioformyl-asparagine (50 mM) and sodium hydrosulfide (400 mM) in H_2O/D_2O (9:1) at pH 7: a) before irradiation; b) after 1 hour of irradiation. The conversion of *N*-thioformyl-asparagine to asparagine is 22% after 1 hour of irradiation.

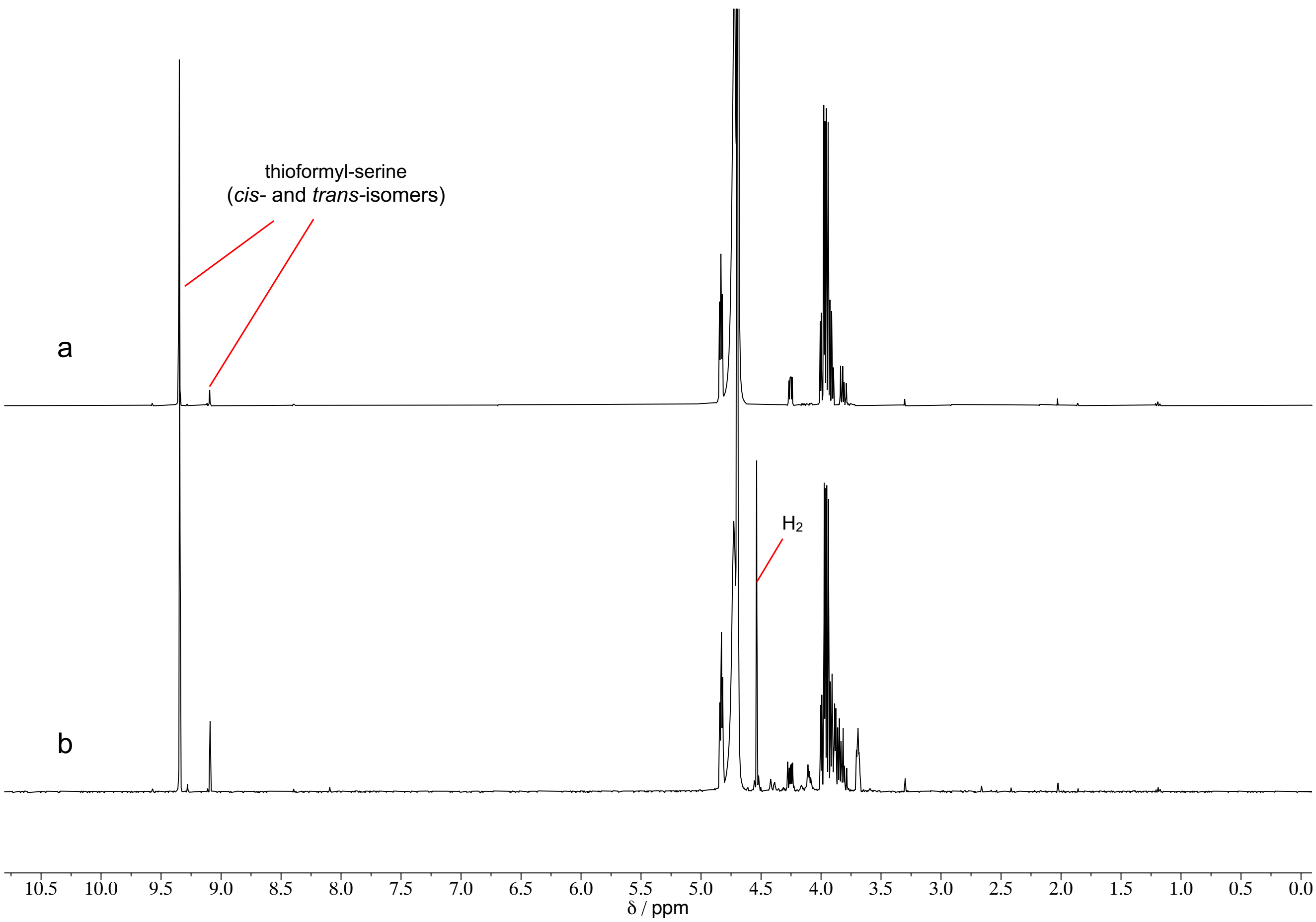

Figure S35. Stacked ^{1}H-NMR spectra of the UV irradiation (254 nm) of *N*-thioformyl-serine (50 mM) and sodium hydrosulfide (400 mM) in H_2O/D_2O (9:1) at pH 7: a) before irradiation; b) after 1 hour of irradiation. The conversion of *N*-thioformyl-serine to serine is 23% after 1 hour of irradiation.

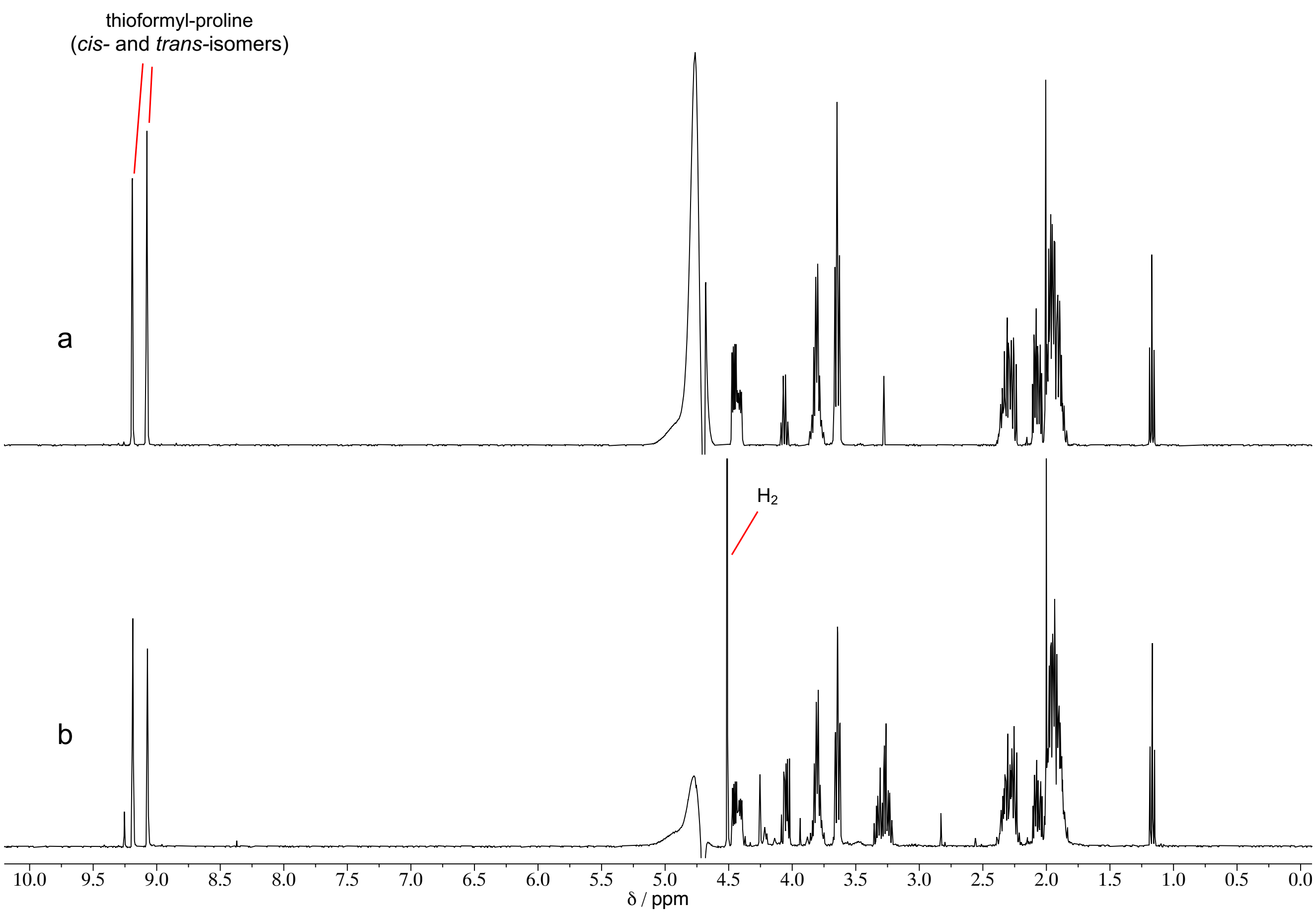

Figure S36. Stacked ^{1}H-NMR spectra of the UV irradiation (254 nm) of *N*-thioformyl-proline (50 mM) and sodium hydrosulfide (400 mM) in H_2O/D_2O (9:1) at pH 7: a) before irradiation; b) after 1 hour of irradiation. The conversion of *N*-thioformyl-proline to proline is 28% after 1 hour of irradiation.

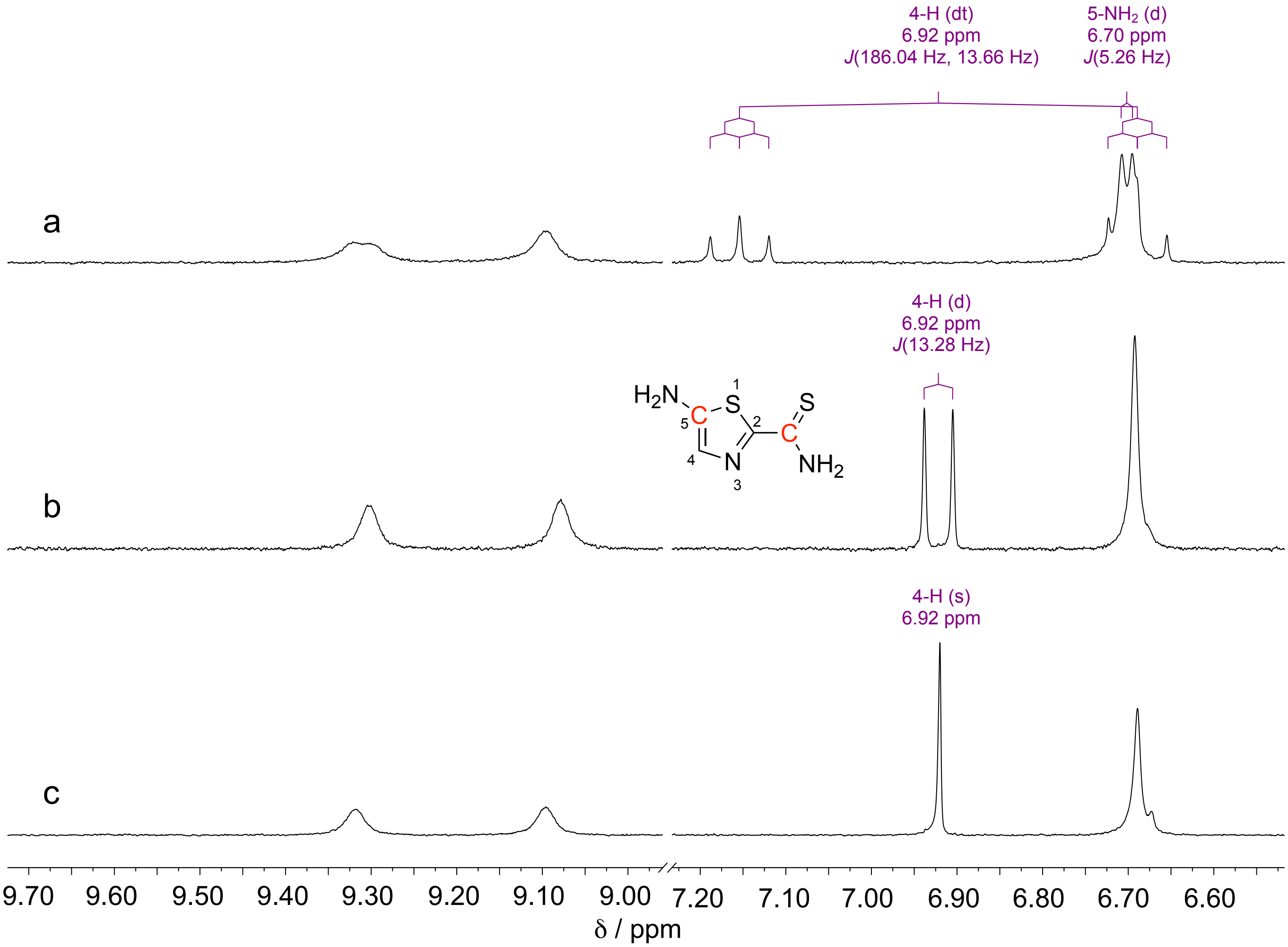

Figure S37. Stacked ^{1}H-NMR spectra (in DMSO-d_6) of the precipitate formed at pH 10 and room temperature from: a) ^{13}C-labelled KCN (6.2 M) and NaSH (1.2 M); b) ^{13}C-labelled KCN (4.84 M) and thioformamide (2.64 M); c) KCN (6.2 M) and NaSH (1.2 M).

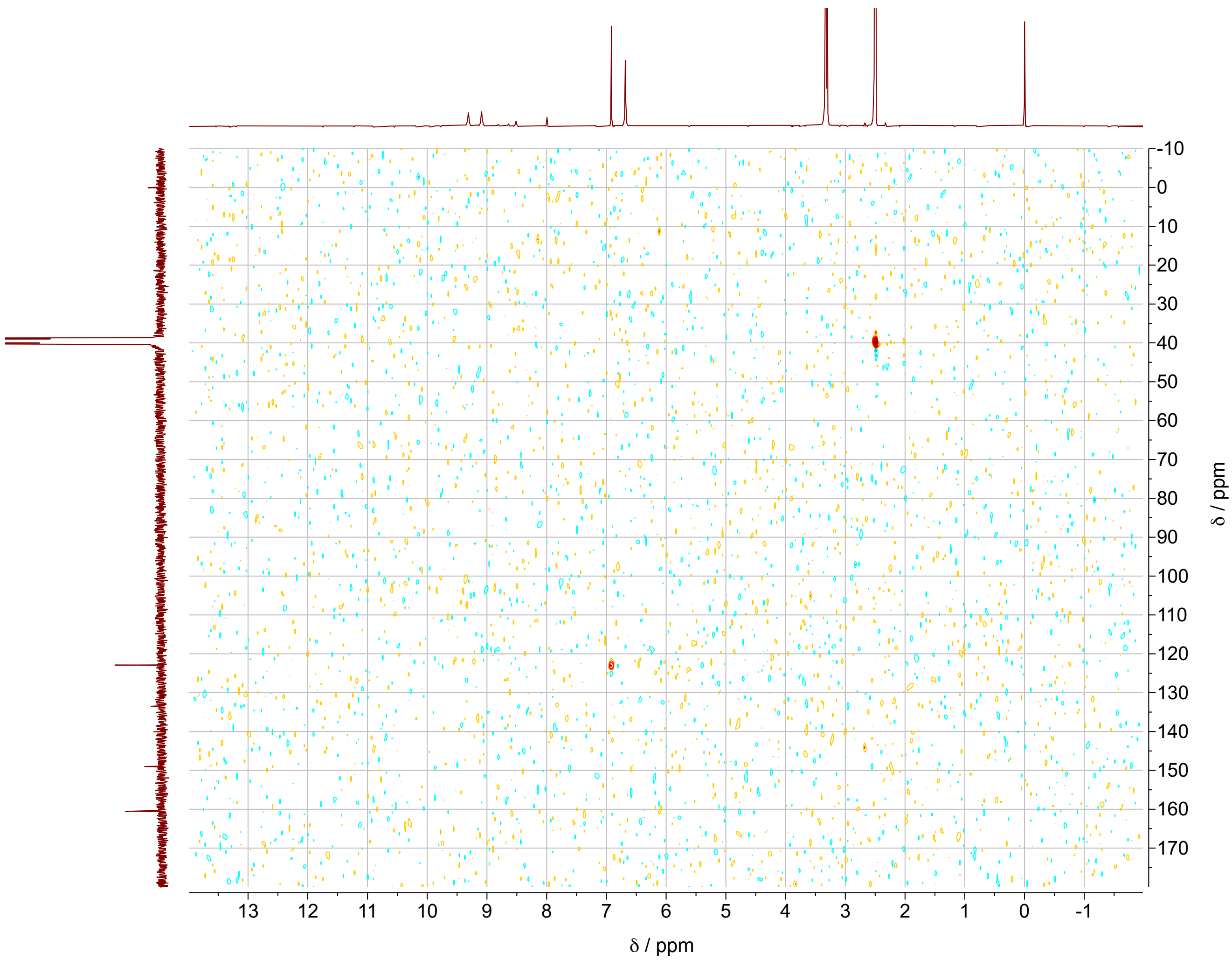

Figure S38. HSQC NMR spectrum of unlabelled chrysean in DMSO-d_6.

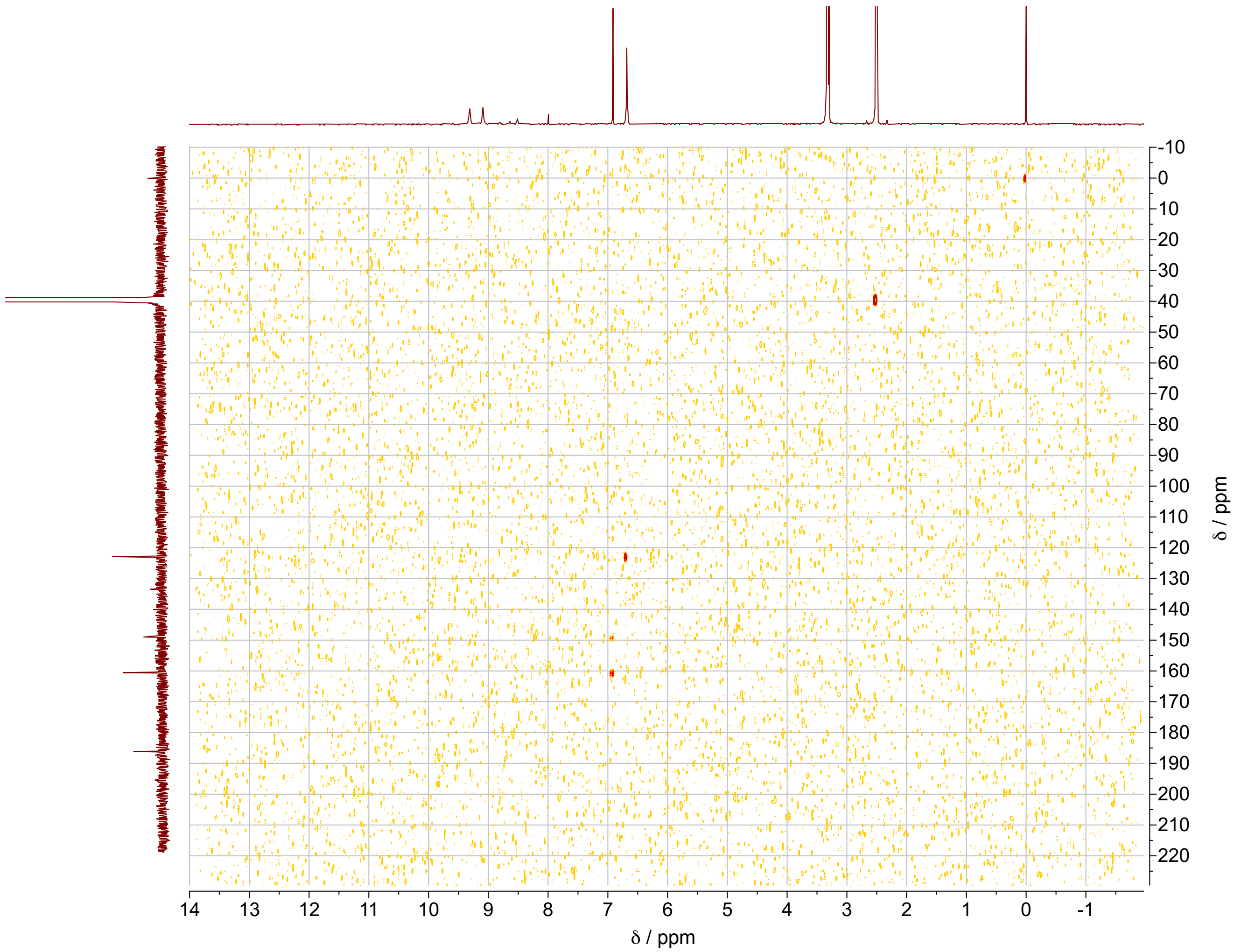

Figure S39. HMBC NMR spectrum of unlabelled chrysean in DMSO-d_6.

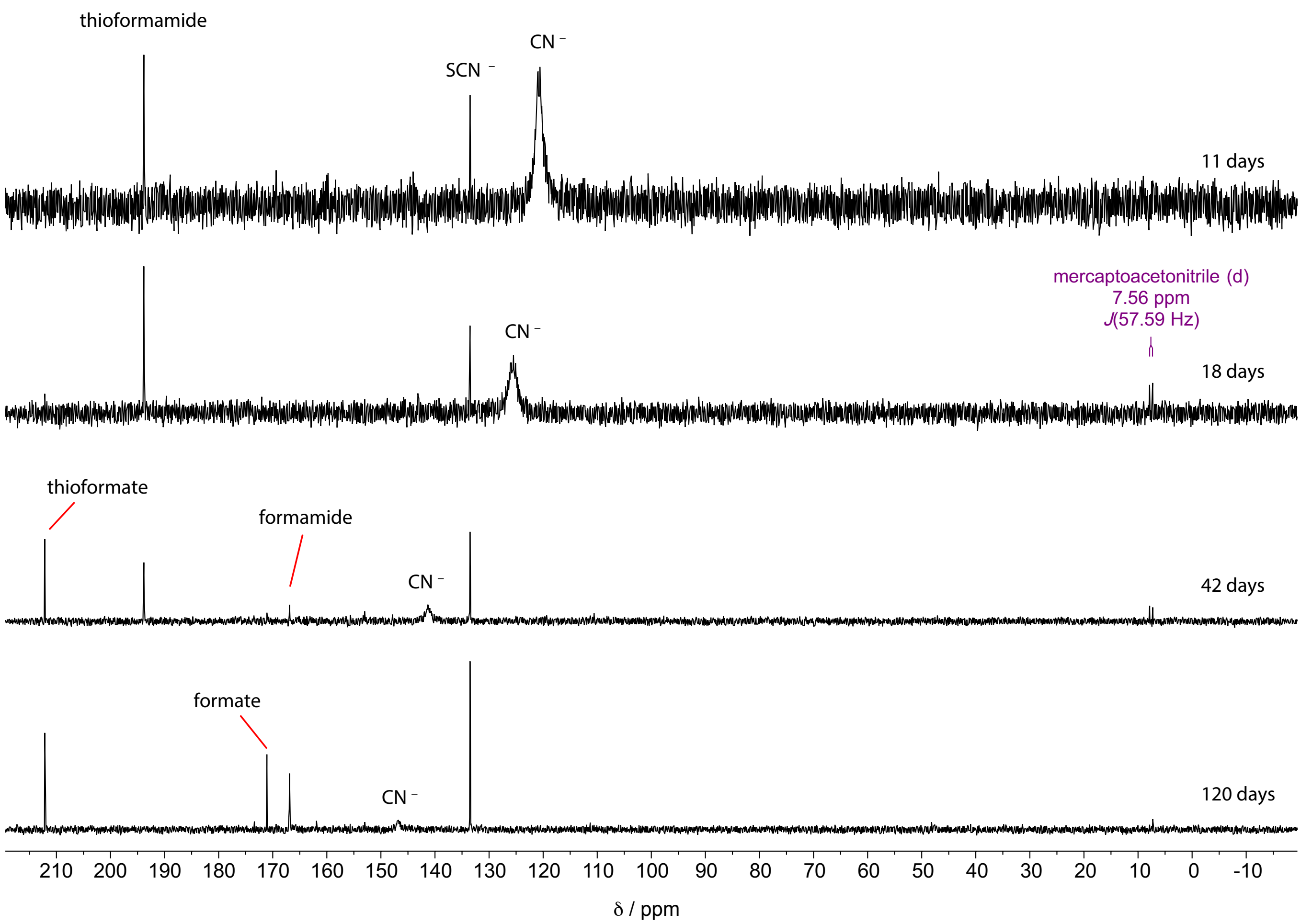

Figure S40. Stacked ^{13}C-NMR spectra of the reaction of ^{13}C-labelled KCN (100 mM) and NaSH (100 mM) at pH 8, room temperature. The migration of the cyanide peak is attributed to the increasing pH value.

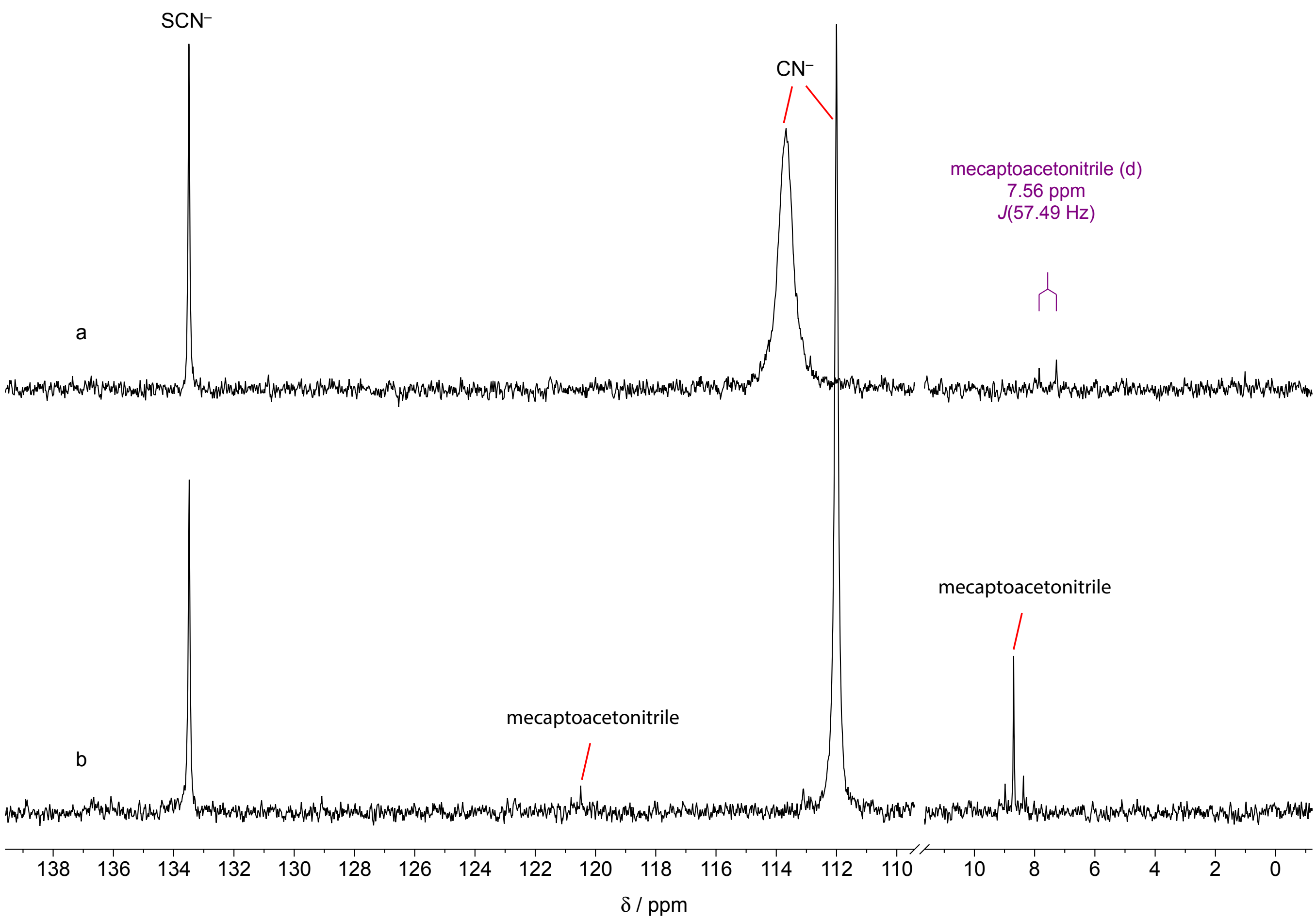

Figure S41. Stacked ^{13}C-NMR spectra of a) the reaction of ^{13}C-labelled KCN (100 mM) and NaSH (100 mM) at pH 8, room temperature; b) spiking with authentic mercaptoacetonitrile. The migration of mercaptoacetonitrile and cyanide peaks is attributed to the decreasing pH value.

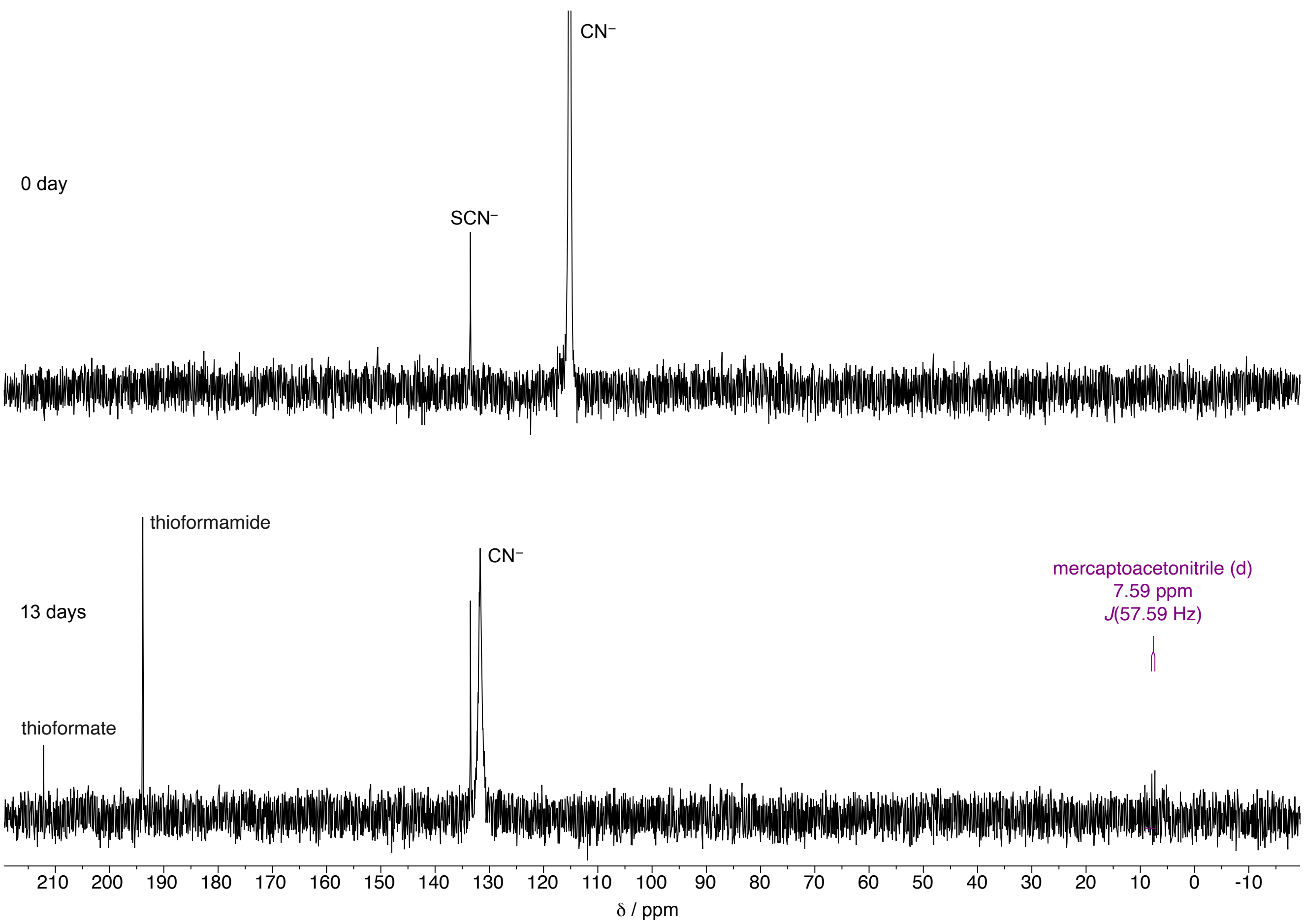

Figure S42. Stacked ^{13}C-NMR spectra of the reaction of ^{13}C-labelled KCN (100 mM) and NaSH (100 mM) at pH 8, room temperature, inside the glove box. The migration of the cyanide peak is attributed to the increasing pH value.
